# Strong Proton‐Phonon Coupling Drives Fast Ion Transport in Perovskites

**DOI:** 10.1002/advs.202507261

**Published:** 2025-12-12

**Authors:** Alexey Rulev, Nobumoto Nagasawa, Hongxin Wang, Vladimir Pomjakushin, Martin Kunz, Yoshitaka Yoda, Stephen P. Cramer, Qianli Chen, Artur Braun

**Affiliations:** ^1^ Laboratory for High Performance Ceramics Empa. Swiss Federal Laboratories for Materials Science and Technology Dübendorf CH – 8600 Switzerland; ^2^ JASRI SPring‐8, 1‐1‐1 Kouto Mikazuki‐cho, Sayo‐gun Hyogo 679‐5198 Japan; ^3^ SETI Institute 339 Bernardo Ave, Suite 200 Mountain View CA 94043 USA; ^4^ Laboratory for Neutron Scattering Swiss Spallation Neutron Source Villigen PSI CH – 5232 Switzerland; ^5^ Advanced Lightsource Lawrence Berkeley National Laboratory Berkeley CA 94720 United States; ^6^ Global College – Shanghai Jiao Tong University 800 Dong Chuan Road Shanghai 200240 China

**Keywords:** activation barriers, fuel cells, phonon DOS, proton conductors

## Abstract

Conduction of protons in solids is a cooperative process propelled by phonons, with molecular details obscured by the irregular movements in the thermal bath. It is shown that substitution with Y forms an imaginary phonon mode, instrumental for the function as proton conductor and effectively lowering the activation barrier for proton transport. To untangle the interplay in the exemplary proton conductor BaSn_0.9_Y_0.1_O_3_, its crystallographic structure is determined with high resolution neutron diffractometry and its phonon density of states with density functional theory calculations, experimentally validated by element specific nuclear resonant vibration spectroscopy. Based on phonon analysis, a quantitative transport model is present, which predicts the activation energy and performance by the ratio of ionic radii. Rather than individual vibrational modes, it is the oxygen sub‐lattice which exerts its momentum on the protons. The extent of this momentum transfer is governed by the ratio of ionic radii. This model extends the transition state theory by the phonon‐phonon interaction and complements the previously proposed idea that lattice dynamics is decisive for proton transport and specifies which properties of the material exactly define the vibration properties.

## Graphical abstract

Experimental and computational phonon analysis of ABO_3_‐type proton conductor BaSnO_3_ shows that substitution on the B‐site with yttrium forms an imaginary phonon mode which is instrumental for the function as proton conductor. This overcompensates the adverse proton trapping effect of the yttrium.

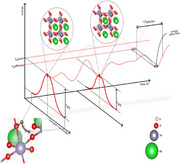

## 1 Introduction

Ion transport is a common process in nature spanning from molecular^[^ ^1^ ^]^ to astronomic^[^ ^2^ ^]^ length scales. In man‐made technology, electrochemical energy storage and conversion are the most important applications of ion transport, such as in batteries, fuel cells and photoelectrochemical cells.^[^ ^3^ ^]^ Ion conducting electrolyte membranes are essential components in solid state electrochemical devices such as high temperature fuel cells (SOFC). Ceramic proton conductors are promising electrolytes for such application.^[^ ^4^, ^5^ ^]^ The desire for improvement of the materials for solid‐state ionic devices is paralleled by the lack of fundamental understanding of their functionalities. The search for the exact nature of the physical driver of the charge transport in such electrolytes is an ongoing quest.

We briefly describe the concept of defect engineering and hydration of the ABO_3_‐type perovskite proton conductors, specifically here barium stannate BaSnO_3_. When the B atom with nominal oxidation state 4+ (here tin Sn^4+^) is substituted with a 3^rd^ group element, for example by yttrium Y^3+^, charge neutrality in BaSn_1‐x_Y_x_O_3‐δ_ (BSYO) requires that oxygen vacancies are formed. Upon exposure to humidity, H_2_O molecules may enter the perovskite structure, the oxygen ion of which can fill the oxygen vacancy. The two protons H^+^ will form O─H groups with adjacent O^2−^ ions.^[^ ^6^ ^]^ At higher temperatures, the O─H bond may break,^[^ ^7^ ^]^ and the H^+[^become mobile species, i.e., protonic charge carriers. The formation of the oxygen vacancies and the hydration of such perovskite materials have been subject of numerous experimental and computational studies, see for example.^[^ ^8^, ^9^ ^]^

A historical model for proton mobility and diffusion, simple but widely established, originally inspired for the case of water, is the Grotthuss mechanism.^[^ ^10^ ^]^ The Grotthuss mechanism illustrates proton conductivity by protons jumping from one oxygen to another over or through the energy barrier. It is generally accepted that the energy barrier is defined by the structure of materials, mostly by repulsive interaction with the B‐atom in ABO_3_ perovskites.^[^ ^11^ ^]^

This model has been adopted for solid materials, specifically for ceramic proton conductors.^[^ ^10^, ^12^, ^13^ ^]^ Proton mobility at the molecular scale has been investigated in detail with experimental and computational methods. For example, quasi elastic neutron scattering on hydrated ceramics has shown that at ambient temperature, the proton rotates around the oxygen ion at high velocity and low activation energy.^[^ ^14^ ^]^ At elevated temperatures, the proton may jump to an adjacent oxygen ion^[^ ^15^, ^16^, ^17^ ^]^ and thus provide net proton conductivity. A recent computational study by Niu et al. investigates the local dynamics of two prominent high frequency vibration modes in yttrium substituted barium zirconate, the O─H stretching mode and wag (reorientation) mode, and their interplay with low frequency proton transport and O─H rotation. They identified three distinct time scales corresponding to local O–H vibration, reorientation, and long‐range proton hopping.^[^ ^18^ ^]^

A contemporary idea is that protons are propelled by specific modes of thermally activated lattice vibrations.^[^ ^19^ ^]^ Vibrational energy can impede or facilitate the transport and conversion of mass and electric charges. Vibration spectroscopy such as infrared and Raman spectroscopy are methods occasionally applied to address this problem.^[^ ^20^ ^]^

We have recently investigated the proton transport in ceramic electrolyte membranes (Y‐substituted barium cerate and zirconate) from ambient conditions to high temperatures and high pressures.^[^ ^21^, ^22^, ^23^ ^]^ We experimentally discovered that the proton diffusion resembles the transport characteristics of a Holstein polaron,^[^ ^24^ ^]^ that is, considering the proton mobility as the random walk as small polarons from site to site, a suggestion previously proposed in a theoretical work by Samgin.^[^ ^25^ ^]^ This observation points to the critical role of vibrations in solids and their underlying structural and cooperative peculiarities, specifically the collective phonon modes. We have previously investigated yttrium‐substituted barium zirconate and barium cerate proton conductors and experimentally found that the activation energy for proton transport decreases when the lattice constant increases (Figure 4‐12 in,^[^ ^26^ ^]^ and^[^ ^22^, ^27^ ^]^). In a targeted follow‐up study, it became clear that the vibration properties affect the proton transport.^[^ ^21^, ^22^, ^28^, ^29^ ^]^ It is therefore of general interest to further explore whether proton transport can be described as a quantum mechanical process and how the crystallographic structure and its characteristic phonon and vibrational behavior play a quantitative role.

Raman spectra were instrumental for our previous discoveries.^[^ ^21^ ^]^ It is a shortcoming of optical vibration spectroscopy that it solely detects phonon modes around the center of the Brillouin zone, whereas the modes that propel ionic transport are considered to be close to the edges.^[^ ^30^ ^]^ We therefore resort to nuclear resonant vibration spectroscopy (NRVS), a rather novel method, which can produce element specific vibration spectra using synchrotron radiation, from molecules and materials, provided those contain Mössbauer active isotopes.^[^ ^31^ ^]^ NRVS has been applied with success, for example, to elucidate the role of metal centers in enzymatic processes.^[^ ^32^, ^33^ ^]^ With NRVS, it is possible to identify the phonon modes of B‐site elements in the BO_6_ octahedra, which are directly related to the proton transport. Because NRVS requires Mössbauer active elements, we consider BaSnY‐oxide as an adequate proton conductor material for fundamental transport studies, with ^119^Sn with 23.87 keV Mössbauer transition energy being the suitable isotope.

Current dopant strategies emphasize how aliovalent substitution modifies crystal structure, defect and proton concentrations, and electrostatic effects such as acceptor‐induced proton trapping.^[^ ^34^ ^]^ Here we show that the dopant's impact on the lattice vibrational properties can be equally, if not more, decisive in setting proton‐jump activation energies. We measured the Sn‐projected partial vibrational density of states (PVDOS) in pristine and Y‐doped BaSnO3 using NRVS. These empirical measurements guided our theoretical approach, which combines density‐functional theory with a fine‐tuned machine‐learning interatomic potential to reproduce the experimental data and subsequently reconstruct the full vibrational spectrum. Leveraging this experimentally validated model, we identified specific oxygen‐dominated modes that dynamically lower the proton‐transfer barrier; these modes soften markedly with Y substitution, leading to increased vibrational amplitudes. This softening is independently consistent with enhanced oxygen Debye–Waller factors extracted from neutron diffraction.^[^ ^35^ ^]^ By correlating the frequencies of these modes with the measured activation energies of proton conductivity, we demonstrate the dominant role of lattice dynamics in governing proton transport and propose a practical framework for incorporating anharmonic vibrations into predictive models of proton conductivity.

## 2 Results

**Figure 1:**
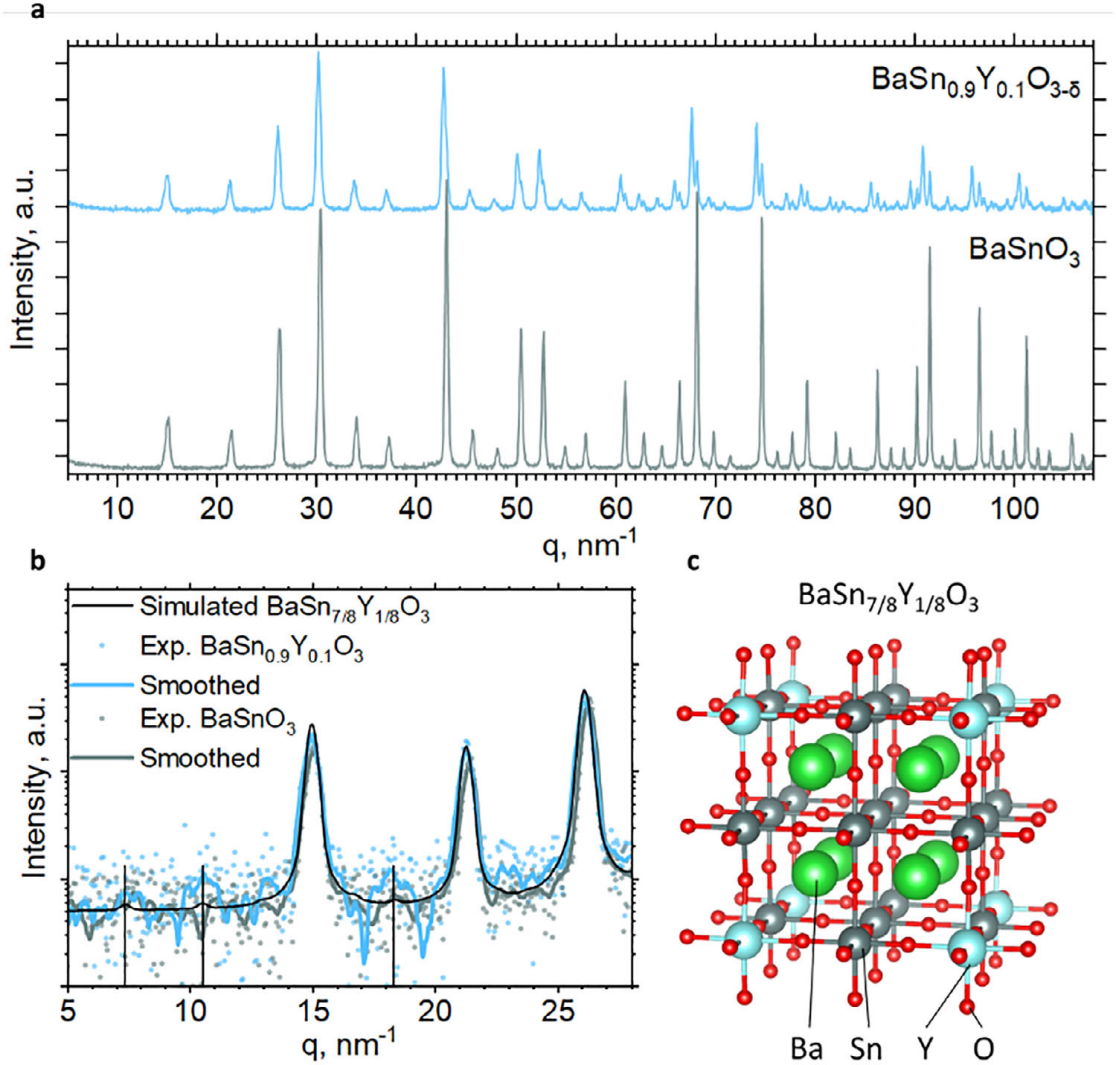
a) Neutron diffractograms of BaSnO_3_ (bottom, gray) and BaSn_0.9_Y_0.1_O_3_ (top, blue). The patterns are offset for ease of comparison. b) Computed neutron diffraction pattern of BaSn_7/8_Y_1/8_O_3_ compared with the experimental data. The extra peaks, originating from superstructure, are highlighted with vertical lines. The Chebyshev polynomial background is subtracted from experimental data. c) Scheme of BaSn_7/8_Y_1/8_O_3_ supercell.

Barium stannate (BaSnO_3_) crystallizes in the Pm3m cubic phase with perovskite structure. Our high‐resolution neutron diffraction data in **Figure** **1** confirm that our sample is a cubic single‐phase perovskite. The lattice parameters determined by Rietveld refinement (see Table S1, Supporting Information) agree with those reported in literature.^[^ ^36^ ^]^ Small deviations from the ideal structure are commonly observed for this material and may originate from minor oxygen or tin deficiency (see for example^[^ ^37^, ^38^, ^39^ ^]^).

The diffractogram of the 10% Y‐substituted sample (BaSn_0.9_Y_0.1_O_3_) in Figure 1 shows a splitting of Bragg reflections at high q‐values. This indicates the existence of two phases in the sample: we can rule out the case of a single‐phase material with lower symmetry, such as I4/mcm, because this did not at all fit the diffractogram.

Table S1 (Supporting Information) shows the lattice parameters for both phases obtained by Rietveld refinement. The first phase generally resembles pure non‐substituted barium stannate, both with respect to lattice parameter and yttrium occupancy, latter of which was equal to zero within the error bar. In the second phase tin is substituted with yttrium and the lattice parameter is slightly larger, which again agrees with published data (for example by Moreira et al.^[^ ^40^ ^]^) and larger ionic radius of Y^3+^. The phase fraction and substitution in the second phase agrees well with total 10% content of yttrium in the sample. The yttrium occupancy in the second phase is also very close to 1/8 (12.5%); this, along with the phase separation, suggests that there may be a superstructure in this phase. Such we found recently for yttrium substituted barium zirconate.^[^ ^41^ ^]^ Draber et al. found by computer simulations that such superstructure can enhance the proton mobility.^[^ ^42^ ^]^ Fransson et al. confirmed by molecular dynamics studies that the softening of an octahedra tilt mode at low temperatures causes superstructure features.^[^ ^43^ ^]^

Figure 1b shows a neutron diffractogram, modelled with a 2x2x2 supercell of BaSnO_3_. There, 1 out of 8 Sn atoms is substituted with Y, which reflects the proposed superstructure. We observe densification of diffracted intensity, i.e., three small peaks that appear due to the superstructure at q ≈ 8, 11 and ≈ 18 nm^−1^; these peaks are present in the experimental pattern, supporting the suggestion that Y forms a superstructure in the material. Along with further analysis detailed in the Supporting Information, there is some evidence that the Y ions in our barium tin oxide may occupy those positions that constitute the superstructure. With the resolution and range of a conventional x‐ray diffractometer, such phase separation and superstructure cannot be distinguished from slight peak broadening and intermediate lattice parameter, which is described in the literature.^[^ ^44^ ^]^ However, our results do not allow us to either confirm or rule out the superstructure and suggest some degree of order parameter, so for further analysis we considered both configurations, with or without superstructure. Also, taking into account the phase separation, we processed the experimental NRVS PVDOS of Y‐doped sample to isolate the contribution of Y‐rich phase from the admixture of the phase without yttrium (Figure S2, Supporting Information)

Using nuclear resonant vibration spectroscopy, as recently proposed by us,^[^ ^45^ ^]^ we can determine the component of the vibration spectrum which originates specifically and only from the ^119^Sn atoms in the proton conductor. We obtain thus the experimental Sn‐projected PVDOS, which we can compare with the calculated Sn PVDOS. This strategy gives us confidence in the entire calculated phonon dispersion reflecting all elements in the material.

**Figure 2:**
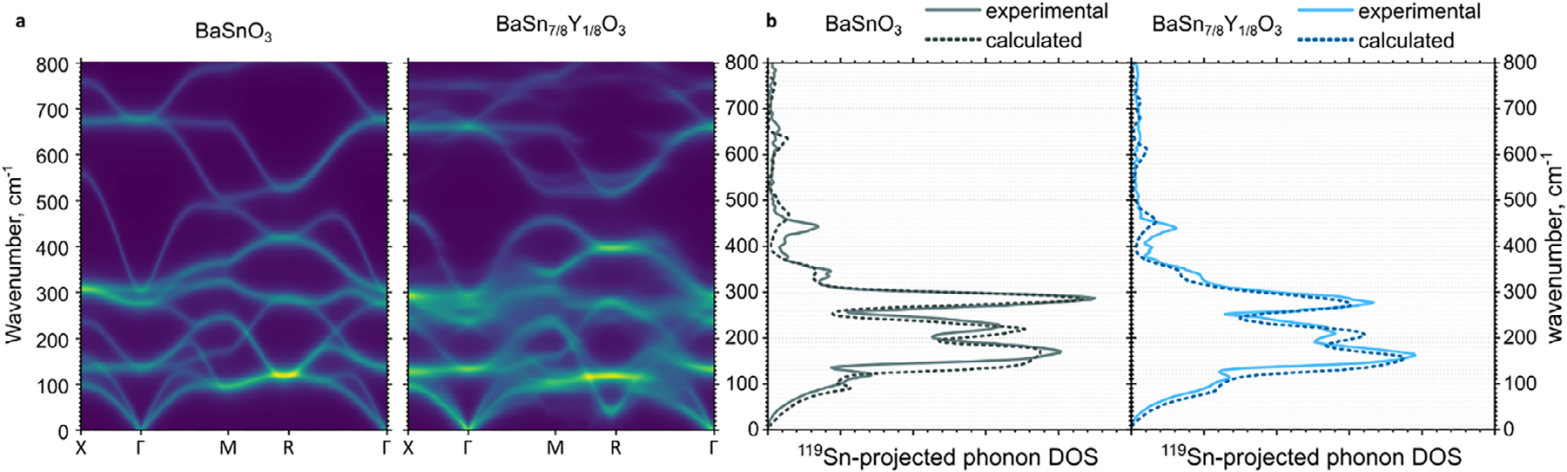
a) Calculated total phonon dispersion curves of BaSnO_3_ and BaSn_7/8_Y_1/8_O_3_. Phonon dispersion curves for BaSnO_3_ were plotted with same spreading (Lorentzian, gamma=1.65 cm^−1^) for consistency. b) Experimental and simulated Sn‐projected phonon DOS of undoped and Y‐doped sample. Gaussian filter is applied to the calculated Sn‐projected PVDOS for comparison with the experimental data. Raw NRVS data are shown in Figures S2 and S3 (Supporting Information).

**Figure** **2** shows two calculated phonon dispersion curves (a) and comparison with the experimental phonon DOS (b): those of pure BaSnO_3_, and those of the separated spectrum of BaSn_7/8_Y_1/8_O_3_. There are noticeable differences between the spectra: the substituted sample demonstrates redistribution of spectral weight of the three major peaks and overall slight shift to lower wave numbers.

To simulate vibration structures of the doped and defect‐containing structures we used machine learning interatomic potential (MLIP) MatterSim, finetuned on the density functional theory (DFT) calculations. The use of MLIP allowed us to simulate ensembles of randomized configurations with defects on larger supercells. In brief, we first tuned some semiempirical parameters for DFT calculations on simple configuration and used that optimized parameters to finetune the MLIP. The simulations on small systems show perfect reconstruction of DFT results with MLIP (Figures S4 and S5, Supporting Information).

**Figure 3:**
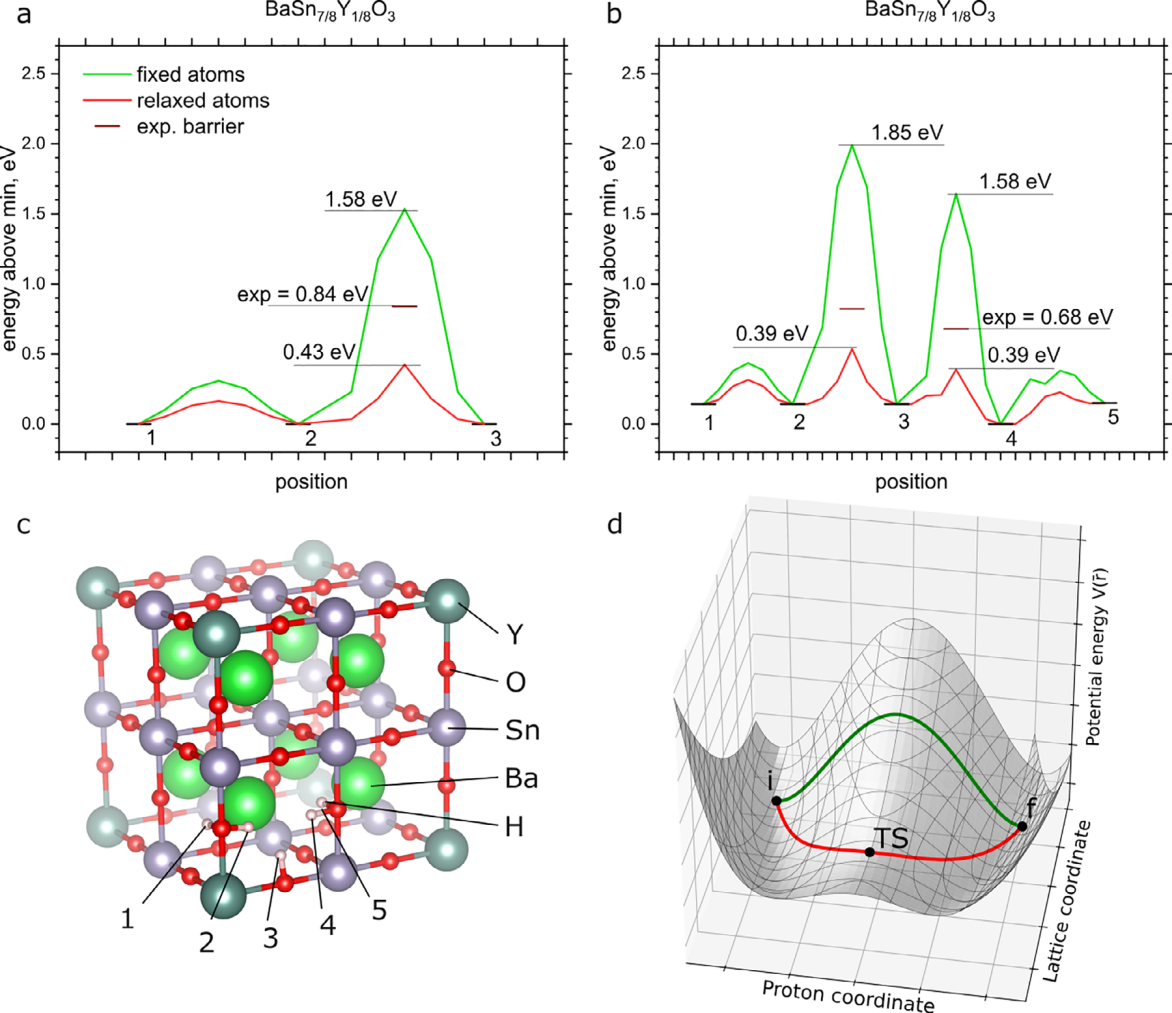
Proton jump barrier in (a) BaSnO_3_ and (b) BaSn_7/8_Y_1/8_O_3_, calculated with NEB method with DFT with all atoms relaxed, or with only proton relaxed, and the experimentally measured values, derived from Arrhenius plots shown in Figure S10 (Supporting Information). Vibrational contribution is not included. Barrier height is indicated relative to the lowest of adjacent equilibrium states. c) scheme of configurations, indicating approximate proton position. d) Schematic potential energy surface $V(\bar{r})$, projected on the plane where one coordinate corresponds to proton position (proton coordinate), and the other to displacements of atoms from equilibrium positions to transition state (Lattice coordinate). Points correspond to initial (i), final (f) and transition (TS) states. Two indicated pathways represent calculations shown in (a).

MLIP simulation of the configurations, containing different number of oxygen vacancies have shown that the best reconstruction of experimental data is achieved with ≈1.2% vacancies for BaSnO_3_ and 2.5% for BaSn_0.9_Y_0.1_O_3_ (see Figure S6, Supporting Information), which is in a good agreement with the experimental data, obtained by Rietveld refinement of neutron diffractograms (as compared to B position occupancy). As we did not manage to reliably validate or rule out the superstructure in BaSn_7/8_Y_1/8_O_3_, we also simulated the configurations with the superstructure. Such configurations reconstruct some features of experimental PVDOS better, and in general have comparable root mean squared error of fitting the experiment; we calculated that the best fit is achieved by a linear combination of PVDOS, produced by superstructured and randomized configurations (weights 0.35 and 0.65, respectively), supporting the idea that the material has some intermediate degree of ordering. However, the difference between these configurations is not very large, so for illustrative purposes and better readability the band structures demonstrated in Figures 2 and **3** were calculated on smaller defect‐free configurations.

DFT with generalized gradient approximation with Hubbard U correction (GGA+U) is essentially semi‐empirical, allowing tuning of some parameters to better simulate real values, in our case interatomic forces. This way, the experimental data served as a reference point to tune both computational parameters and configuration of simulated cells. Following the Grotthus mechanism, vibrations of oxygen should be of primary interest in relation to proton conductivity; whereas the vibrations detected with NRVS, however, are related to tin (^119^Sn) atoms only. But in crystalline material, the entire set of vibrations is related to each other via the interatomic forces which maintain the structure. If we can simulate the Sn‐projected PVDOS good enough that it matches the NRVS based experimental spectrum due to ^119^Sn, then we have a reference point and confidence that the whole vibration spectrum originating from all ions in the unit cell (also those ions not probed by NRVS: Ba, Y, O) is simulated with the same accuracy. Previously we have demonstrated that the accurate simulation of Mössbauer‐active element‐projected vibrational DOS also results in precise match of all other vibrational frequencies, measurable with, for example, Raman spectroscopy.^[^ ^46^, ^47^ ^]^ Thus, good agreement between the experimental PVDOS and the one calculated with MLIP gives us confidence in the correct simulation of real interatomic forces that govern all vibrational properties. It then translates to DFT, which was used to finetune the MLIP, that we use for further detailed analysis.

Ionic conductivity σ is the product of proton concentration and mobility. In hydrated ABO_3_ perovskites, oxygen vacancies are filled by oxygen from water, producing O–H groups and mobile protons.^[^ ^48^ ^]^ Substituent choice also governs hydration potency and proton concentration (e.g., Sc vs Y in BaZrO_3_) and can introduce acceptor–proton association (“trapping”), typically expressed as an association energy, that is supposed to hinder conductivity.^[^ ^49^, ^50^, ^51^ ^]^ Here we focus on mobility, that is usually characterized by activation energy, which is the potential energy barrier for a proton jump between adjacent oxygen atoms. A straightforward calculation of the barrier height with DFT using nudged elastic bands method (NEB) leads to a significantly underestimated barrier height, when compared to the experimentally measured values. The potential energy barrier is 0.43 and 0.39 eV for BaSnO_3_ and BaSn_7/8_Y_1/8_O_3,_ respectively (red line in Figure 3a,b), and becomes 0.39, and 0.25 eV after the vibrational contribution is included according to the (variational) transition state theory (TST)^[^ ^52^ ^]^ (see Table S2, Supporting Information), while the experimental values, that we derived from Arrhenius plots (Figure S10, Supporting Information) are 0.84 and 0.68, respectively (Figure 3), both in agreement with previously reported results^[^ ^53^ ^]^ (although BaSnO3 is not acceptor doped, it still has some proton conductivity due to partially reduced Sn^2+^, see discussion in Supporting Information).

Calculations of proton mobility using the NEB method are known to significantly underestimate the barrier height compared to experimental values.^[^ ^54^, ^55^, ^56^, ^57^ ^]^ This discrepancy is typically rationalized as electrostatic “proton trapping” by the acceptor dopant. In our study, we challenge this interpretation, demonstrating that an alternative mechanism better accounts for the observed behavior, particularly given the accuracy with which DFT can model electrostatic interactions.

In addition, this can obviously not be the case for the undoped BaSnO_3_. If the barrier is calculated with the lattice atoms fixed at their positions at equilibrium (and interpolated in intermediate images), the barrier becomes significantly larger (green lines in Figure 3a,b), indicating that lattice relaxation plays a significant role in this process. We propose that such failure originates from severe anharmonicity of proton‐related vibrations, that leads to incorrect results by the practical approximations with TST.

Briefly this anharmonicity can be pictured as following: in the oxide crystal proton exists as a hydroxyl group OH, with strong bond energy and definite length. The fact that vibration frequency of this bond is almost one order of magnitude higher than frequencies of lattice vibrations (typically ≈100 THz or ≈3300 cm^−1^,^[^ ^58^ ^]^ compare to lattice vibrations in Figure 2; Figure S6, Supporting Information) means that the proton has equilibrium position relative to the “slowly” vibrating oxygen atom, and not to some definite position in the unit cell, as it should be in harmonic approximation.

We can project a 3(N+1) – dimensional potential energy surface (PES) (N – number of lattice atoms and one proton) on the 2D plane, which first coordinate is pointed along the proton position from one equilibrium position to the other, and second coordinate points along the displacement of N lattice atoms from equilibrium positions toward the saddle point or transition state (TS). A doodle of such projection is shown in Figure 3d, where horizontal axis corresponds to proton coordinate, and the vertical axis to all lattice atoms. Although the displacement of the proton is obviously larger than displacement of the lattice atoms, if the plane is presented in isoinertial coordinates, that are inversely proportional to the square root of mass,^[^ ^52^ ^]^ the displacements of lattice become comparable, if not larger, to the displacement of the proton, which has a low mass (note that here we consider movement of many lattice atoms at once, not one single oxygen atom). This feature leads to large shift of the TS position from a straight line, connecting two equilibrium states. With this information we can outline where the approximations of TST fail to estimate the barrier height. As formulated by Vineyard, the transition rate Г (jump rate in our case) is defined as ratio of systems that cross the dividing surface, marked by the dashed line in Figure 3, (I) to the total number of systems on one side of the surface (Q_i_)^[^ ^59^ ^]^:

(1) $$\Gamma =\frac{I}{Q_{i}}$$

This expression does not include any assumptions yet (except for recrossings, which is beyond the scope of our paper) and should stand for all systems, including anharmonic. To calculate the practical values like activation energy and pre‐exponential factor we need to apply harmonic approximation and calculate all the vibrational frequencies at the equilibrium point A, transition state and the difference in potential energy between these points. Essentially that means that we calculate how populated is the 3N‐dimensional paraboloid at TS for numerator, and 3(N+1)‐dimensional one at equilibrium position. Application of harmonic approximation results in the following equation for the transition rate:

(2) $$\Gamma =\frac{\prod_{j=1}^{3\left(N+1\right)}\nu {\left(i\right)}_{j}}{\prod_{j=1}^{3\left(N+1\right)-1}\nu {\left(TS\right)}_{j}}e^{\frac{V\left(i\right)-V\left(TS\right)}{kT}}$$

where V(i) or V(TS) are potential energies of initial state and saddle point, respectively, k is Boltzmann's constant, T the absolute temperature and the pre‐exponential factor numerator (effective attempt frequency) is a product of all normal frequencies of vibration *ν(i)* around the initial state *i*, denominator is product of all normal frequencies of vibration *ν(TS)* around the initial state *TS*, except for one imaginary frequency corresponding to the saddle point. Since the frequency of O─H bond vibration is substantially larger than other frequencies of lattice vibration, it dominates the pre‐exponential factor, and effective frequency becomes approximately equal to the relatively high frequency of O─H bond vibration. This means that in this approximation the systems from the neighborhood of point i in Figure 3d are supposed to perform jump attempts to overcome barrier of height V(TS)‐V(i) (along red line), but at a frequency that approximately corresponds to movement along the green line, which is obviously not correct. Therefore, to generalize the idea, the deviation of one pathway from another is the main cause for the issue.

To demonstrate where this discrepancy happened, we can go back to Equation 1. Since in the TS the proton‐related coordinate, that brings most anharmonicity, is dropped, the harmonic approximation for the numerator is well suited. However, it is not true for the denominator: one can clearly see that if we calculate number of systems Q_i_ by approximating PES around initial state with a paraboloid, the result will severely underestimate real Q_i_ value, since PES is stretched toward the TS. This underestimation of the denominator leads to overestimation of the transition rate and hence, underestimation of the activation energy. Notably, that this effect becomes stronger if the TS is shifted more from the straight line, so it is most expressed for very light ions such as protons.

To the best of our knowledge, there is no existing framework to circumvent this issue. Here we propose a metric that can be calculated from the NEB simulations and phonon structure of the host lattice. The approach allows us to estimate how the vibrations would influence the barrier height and predict the activation energies for different materials with similar chemistry and structure.

**Figure 4:**
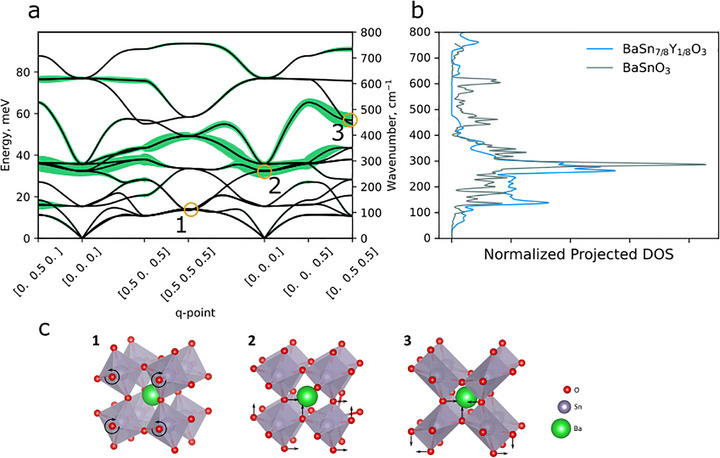
a) Phonon dispersion in BaSnO_3_ and the squared length of projection of atomic displacement onto phonon eigenvectors (b) calculated projected density of projected phonons eigenvalues on the displacement of lattice atoms from equilibrium to transition state in BaSnO_3_ and BaSn_7/8_Y_1/8_O_3_; c) schematic representation of the displacements, corresponding to some modes indicated in panel (a).

For that we calculated the vector of displacement of all atoms except for proton from initial and final state toward the transition state that corresponds to the vertical axis in Figure 3d. Then we calculate length of projection of a phonon eigenvector onto the displacement vector; this way we estimate how much vibration along this mode drives the system toward the transition state. In this case we took phonon eigenvectors, calculated in ideal system that doesn't contain protons, onto displacements of all atoms except for the proton, calculated in proton‐containing system. This way we isolated the effect of lattice vibration. **Figure** **4**a demonstrates squared lengths of such projection for different phonon modes in BaSnO_3_, and the pattern of atomic displacements, corresponding to the modes. We decided to plot square of the length since we try to estimate its influence on the energy barrier, and energy in first approximation is proportional to the square of displacement. In this calculation we considered proton jump in a‐c plane, corresponding to the jump 2‐3 in Figure 3c. It can be easily seen that the modes that bring involved oxygen atoms closer (for example, modes 2 and 3), have higher projection lengths, than the modes that are indifferent for proton jump length (mode 1). Summarizing, by calculating the projection length we now can highlight specific phonon modes that dynamically lower the proton jump barrier.

Figure 4b demonstrates density of such projections as a function of phonon wavenumber in BaSnO_3_ and BaSn_7/8_Y_1/8_O_3_. On average, the frequency of the modes with large projections in the Y‐doped sample is lower than in the undoped. The measured activation energy in Y‐doped sample was notably lower, what correlates well with lower frequency of related phonon modes. In our presentation it means that the lattice can vibrate in the direction of the transition state at higher amplitude, allowing the proton to perform jumps at effectively lower barrier. This concept is also in good agreement with the previous data that associates higher activation energy with phonon hardening.^[^ ^22^ ^]^

**Figure 5:**
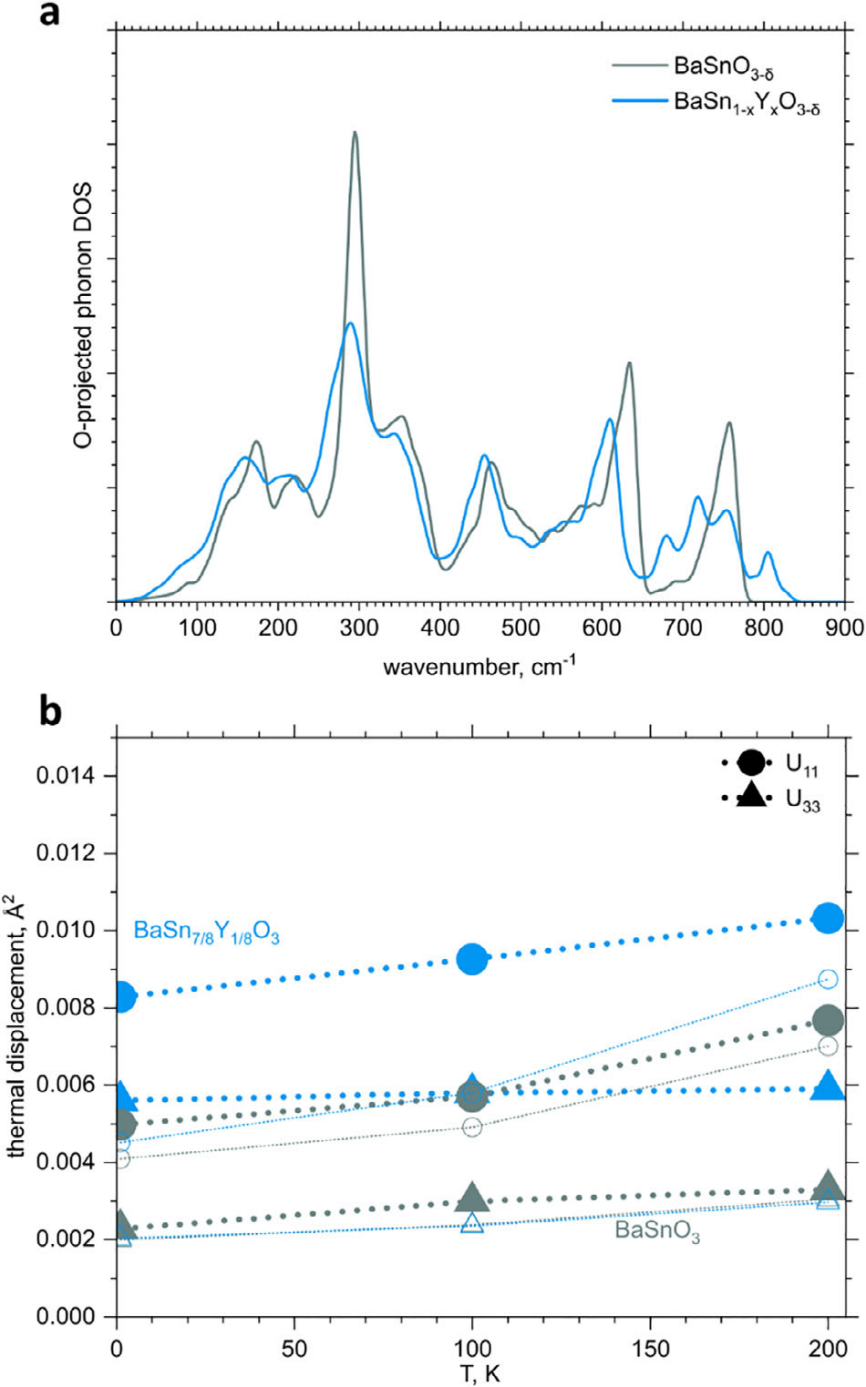
a) Calculated oxygen projected phonon density of states of BaSnO_3_ and BaSn_7/8_Y_1/8_O_3_. b) Anisotropic thermal displacement of oxygen atoms, obtained from Rietveld refinement of neutron diffraction data, and the calculated values. U_ij_ corresponds to matrix elements of the thermal displacement tensor: U_11_=U_22_ corresponds to displacement perpendicular to the Sn─O─Sn(Y) line, U_33_ – along that line. Filled symbols correspond to experimental data, open – calculated data.

Naturally these modes are mostly associated with oxygen atoms (see,^[^ ^46^ ^]^ Figure 3). Within the harmonic approximation the phonon frequency ω is proportional to the second derivative of the potential energy surface along the displacement of relevant atoms:

(3) $$\omega \left(\bar{r}\right)∼\frac{\partial^{2}V}{\partial {\bar{r}}^{2}}$$

where V is the potential energy of the system and $\bar{r}$ are generalized atomic coordinates. The lower phonon frequency in the Y‐substituted sample means that there is a flatter potential energy surface around the equilibrium and hence, larger amplitude of vibration at the same temperature, as follows from the expression for the mean square atomic displacement (Ch. 4 in^[^ ^60^ ^]^):

(4) $$\langle {\left|Q_{\nu }\left(k\right)\right|}^{2}\rangle =\frac{\hbar }{\omega }\left(n_{\nu }\left(k,\omega ,T\right)+\frac{1}{2}\right)$$

where the left side is the thermally averaged squared normal mode coordinates for a mode ν with a wavevector k, and n(ω,T) is mode occupation number, expressed as:

(5) $$n_{\nu }\left(k,\omega ,T\right)={\left[\exp\left(\frac{\hbar \omega_{\nu }\left(k\right)}{k_{B}T}\right)-1\right]}^{-1}$$

which also monotonically decreases with ω. Mostly phonon modes involving oxygen have lower frequency in a substituted sample, as seen in the oxygen‐projected PVDOS (**Figure** **5**a) and that should result in the overall larger thermal displacement of oxygen atoms in the Y‐substituted sample. We have determined the average thermal displacement from the Rietveld refinement of our high‐resolution neutron diffraction data. The values of anisotropic thermal factors for oxygen atoms are shown in Figure 5b, along with the simulated values. All matrix elements for the thermal displacements of oxygen in the substituted sample are approximately 1.5 to 2 times larger than for the unsubstituted sample. Partially this increase may be attributed to the structural disorder caused by Y doping, that is indistinguishable for diffraction from thermal displacement and leads to larger deviation of calculated parameters in case of Y‐doped sample. But the significant contribution is due to lower frequency of oxygen vibrations, which is supported by atomistic modelling. Further increase of the atomic displacements upon temperature increase to 100 K, when phonon states with wavenumbers up to ≈70 cm^−1^ are mostly occupied according to Equation (3), is larger for Y‐doped sample. That reflects the feature at 30–80 cm^−1^ shown in Figures 4b and 5a, thus confirming the vibration data and validating our simulations. Thus, results of neutron diffraction serve as another experimental validation of the calculated results, shown in Figure 4b.

Besides the minor differences in the band structure, such as band broadening due to lower symmetry of the structure, one of the main differences is in the frequencies of some modes at M‐ and R‐points of a Brillouin zone. In the Y‐substituted sample, the lowest energy band at the R point is shifted on the frequency axis to an energy almost two times lower than in the unsubstituted sample. These bands correspond to distortions or tilt of the oxygen octahedra, as illustrated in the crystal structures in Figure 4. Such octahedral tilts are very closely associated with the structure of perovskites, which can have static octahedral tilts, if frequency of some modes at the edge of Brillouin zone becomes imaginary. The relations between ionic radii of A and B atoms and octahedral tilts are comprehensively studied and well understood,^[^ ^61^, ^62^ ^]^ what makes perovskites family a perfect model system to demonstrate the described phenomena. This all allows us to establish and demonstrate relatively simple relations between crystal structure, vibrational structure and their ionic conductivity.

In perovskite materials the tendency for such tilt is quantified by a structure tolerance factor. The structure tolerance factor, in the simplest form the Goldschmidt tolerance factor *t*, is defined as follows:

(6) $$t=\frac{r_{A}+r_{O}}{\sqrt{2}\left(r_{B}+r_{O}\right)}$$

The tolerance factor points to the limits when the material will have ideal cubic structure, or undergo certain structural distortion.^[^ ^61^ ^]^ Other more detailed expressions for the tolerance factor are also proposed,^[^ ^62^ ^]^ but in general they all have monotonic relationship with *r_B_/r_A_* ratio (namely, monotonic decrease). Since the structural distortion is set directly by the potential energy surface for oxygen atoms, we can place the materials along the same ratio *r_B_/r_A_*, as demonstrated in Figure 5. We chose this parameter as the ordinate to preserve linear dependence from the *r_B_*, when *r_A_* and *r_O_* are constant, in an attempt to maintain linear relations similar to the concept of Vegard's rule.^[^ ^64^ ^]^ This parameter was also chosen in a phase diagram which separates the itinerant from localized electronic systems in perovskites, which is known as “Nakamura field map”.^[^ ^65^, ^66^ ^]^ The effective atomic radii were taken from ref. [67]; for the doped systems, weighted average ionic radius was taken.

**Figure 6:**
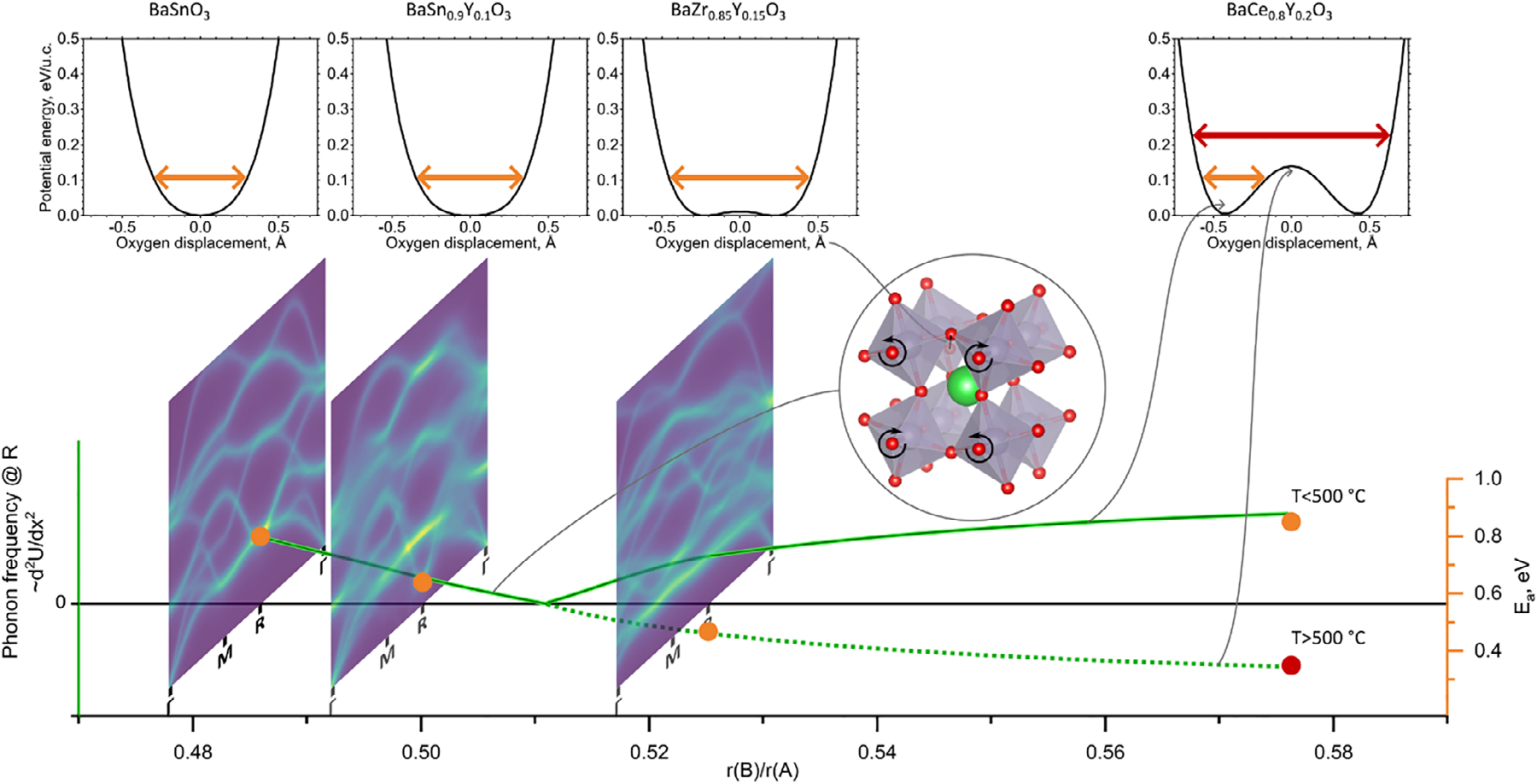
Influence of the structure on the potential energy landscape, phonon frequency and proton conductivity activation energy as function of atomic radius ratio. Activation energies were: Ea(BaSnO_3_) = 0.84 eV, Ea(BaSn_0.9_Y_.01_O_3_) = 0.68 eV (this paper), Ea(BaZr_0.85_Y_0.15_O_3_) = 0.467 eV,^[^ ^50^ ^]^ Ea(BaZr_0.85_Y_0.15_O_3_) = 0.85 eV below ≈ 773 K, 0.35 eV above 773 K.^[^ ^63^ ^]^ The animations of vibration modes are shown in Video S1 (Supporting Information).

We can trace the frequency of a phonon mode, corresponding to the octahedral tilt (a^0^a^0^c^−^ in Glazer notation^[^ ^68^ ^]^) along the vertical axis (abscissae), and trace the frequency along the composition range (ordinate). As we mentioned earlier, the second derivative of the PES along the phonon mode is proportional to the frequency of the mode, so it should follow the same trend. When this line crosses zero, the PES at 0 (i.e., when oxygen atoms rest on the line connecting two B atoms) should have negative second derivative, so the PES would have a saddle point at 0 and minima somewhere aside.^[^ ^63^ ^]^ We observe this in Figure 5 as a bump at zero oxygen displacement for Y‐doped barium zirconate or cerate. This means that statically the structure would tend to be distorted, so the intercept point where the frequency, or the curvature of PES, crosses zero would correspond to the structure tolerance limit, beyond which the distortions may be observed. For example, in barium cerate, which is far beyond the structure tolerance limit,^[^ ^69^ ^]^ calculation of phonon frequencies in cubic symmetry results in imaginary phonon modes, indicating that there is a saddle point. In the distorted structure, all the frequencies are again positive (as shown for yttrium‐doped barium zirconate in **Figure** **6**), so we can trace this frequency in the graph, which would be proportional to the curvature of PES around this minimum.

Such phonon modes with imaginary frequencies (plotted as negative)^[^ ^70^ ^]^ are often indicative to dynamically instable systems.^[^ ^71^ ^]^ As a side note, such dynamically instable systems often present peculiar charge transport properties. For example, in the Nakamura field map^[^ ^65^, ^66^ ^]^ for ABO_3_ perovskite materials it is observed that the materials located near the borderline between itinerant and localized electron systems, happen to be the “good” cathode materials for solid oxide fuel cells.^[^ ^72^ ^]^ It is also noteworthy that superconductivity may originate from presence of such imaginary phonon modes, as for example found in Y_2_C_3._^[^ ^73^ ^]^ Superionic conductivity in Li_3_N appears to originate from imaginary phonon modes.^[^ ^74^ ^]^ Gupta and co‐authors have investigated several ionic conductors, Na_3_PS_4_,^[^ ^75^ ^]^ Na_3_FY (Y = S, Se, Te)^[^ ^76^ ^]^ and Cu_7_PSe_6_^[^ ^77^ ^]^ and come to similar conclusions.

We note that we selected this specific phonon mode for illustrative purposes. Although this mode itself doesn't lower the barrier (Figure 4), from one side, it has a very straightforward connection to structure and from the other is deeply correlated with other oxygen‐related modes. Perovskite structures may undergo a variety of distortions that can be related to different phonon modes. In addition, as the main proton transport pathway, the proton jumps between two oxygen atoms along the octahedron edge, the length of which is unaffected by this vibration. The PES shown in Figure 6 corresponds to the simultaneous movement of all the oxygen atoms in the lattice in various directions; calculations show that the PES for individual movement of the oxygen atom follows the same trend (Supporting Information, Figure S7, Supporting Information), and this is also supported by experimental data (thermal displacement parameters from ND). Therefore, we use the frequency of this mode only as a manifest feature; in general, one should perform evaluation similar to the one shown in Figure 4 and somehow quantify the density of the projections, but in perovskite materials they are correlated, so we use this frequency as a proxy.

As we have proven earlier for the barium stannate and Y‐substituted stannate, the curvature of the PES is inversely proportional to the atomic displacement of the oxygen atoms, which in turn is inversely proportional to the activation energy of proton jump, given that the “static” lattice parameter does not significantly change. Therefore, the activation energy should follow the same trend (compare Equation 6 in^[^ ^24^ ^]^), like the frequency of the considered phonon mode, although we do not imply a specific functional relation.

Figure 6 shows the overlaid activation energy in different materials. For the barium stannates, experimentally investigated in this work, the activation energy follows this trend and decreases with increasing ratio *r_B_/r_A_*. Just after passing the structure tolerance limit, the so‐called soft phonon modes are observed. Notably, a higher *r_B_/r_A_* ratio and phonon softening indicate a larger volume of the oxygen octahedra and larger oxygen–oxygen separation. However, the activation energy decreases with an increasing *r_B_/r_A_* ratio, highlighting that the proton mobility is not governed by the static lattice structure, but the dynamic phonon motion. Imaginary phonon modes are often related with softening of phonon modes,^[^ ^71^, ^73^, ^74^ ^]^ consistent with the observation that when adding Y in BaSnO_3_, the phonon frequency is lower. These imaginary phonon modes have low imaginary frequency, indicating a very shallow saddle point at the potential energy landscape, as was calculated for barium zirconate. Such shallow saddle point means that oxygen atoms can easily overcome this barrier and vibrate between the minima on the side, being on average in the middle and resulting in apparent cubic symmetry. Experimental data for barium zirconate show that it has cubic structure, although calculations show that it is beyond the tolerance limit. This is in agreement with the finding that in barium zirconate, oxygen atoms have anomalously high Debye‐Waller (thermal displacement) factors,^[^ ^78^ ^]^ so we can state that the displacements still follow the bottom red line in Figure 4, as does the measured proton conductivity activation energy (Ea(BaZr_0.8_Y_0.2_O_3‐δ_) = 0.467 eV,^[^ ^50^ ^]^ Ea(BaZr_0.85_Y_0.15_O_3‐δ_) = 0.442^[^ ^79^ ^]^). Thus, this bottom line not only describes the curvature of the PES at 0, but also the breadth of the PES above the saddle point.

If the barrier is sufficiently high, so that oxygen atoms below a specific temperature cannot overcome it and thus vibrate around the local minimum, then the thermal displacement of oxygen atoms would follow the line, corresponding to the curvature of the PES around the minimum. Thus, the amplitude of thermal displacement of oxygen starts decreasing, and the activation energy for proton conductivity again increases, what was indeed observed in the experiment (Ea(BaCe_0.8_Y_0.2_O_3_) = 0.85 eV^[^ ^63^ ^]^).

However, this barrier can still be overcome above that specific temperature. It was observed that Y‐substituted barium cerate undergoes a phase transition to higher symmetry at around 773 K that is accompanied by the significant decrease of the activation energy (Ea = 0.35 eV).^[^ ^63^ ^]^ The model that we present allows to explain this decrease by the fact, that this phase transition means switching to vibrations in the much wider range through the saddle point. We can state that while below this temperature thermal displacement amplitude corresponds to the top line in Figure 6, above 773 K the displacement corresponds to the lower line. The activation energies, measured for this material, perfectly follow these trends that have thermal vibrations in a foundation. It indicates that the thermal vibrations and phonon structure are the most important properties, defining the proton transport activation energy. This means that proton transport in particular (and possibly ion transport in general) are cooperative processes of proton‐phonon coupling, where the entire crystal lattice acts on the proton by momentum transfer.

## 3 Discussion

Previously it was found that larger lattice volume correlates with lower proton jump activation energy.^[^ ^12^, ^41^ ^]^ That agrees with our previous results that shown that the proton conduction activation energy in Y substituted barium zirconate and cerate scales reversely with the lattice parameter.^[^ ^21^, ^22^, ^26^, ^29^ ^]^ It is in contrast to the calculations by Münch^[^ ^80^ ^]^ and Marx^[^ ^10^ ^]^ who find that the barrier height decreases with decreasing oxygen–oxygen separation. This counterintuitive situation can be resolved by understanding that the displacements of the oxygen ions upon thermal excitation become larger and their relative distances occasionally become shorter, particularly when substituted with yttrium. While such scenarios have been suggested by Kreuer,^[^ ^13^, ^81^ ^]^ Münch^[^ ^80^ ^]^ and Marx,^[^ ^10^ ^]^ we provide here evidence from experiment and calculation that probability of proton transfer depends on the soft phonon modes formed by the yttrium ion, which contributes a desirable transport function to the crystal.

Here we have shown that this might be so not because the proton per se is freer to move in such large volume lattices; rather, oxygen atoms, to which the proton is attached, are freer to vibrate within the lattice with a large unit cell volume. For example, in aluminum oxide, which has a small unit cell volume, the activation energy is rather large, not because of the small volume, but because of the higher energy of oxygen‐projected phonons in aluminum oxide,^[^ ^82^ ^]^ compared to the perovskites.

Our results show that adding Y in BaSnO_3_ induces phonon softening, which shifts the phonons that influence the barrier height to lower frequencies. The phonon softening overcompensates the proton trapping effect originating from the electrostatic interaction and local lattice distortion of Y and improves the proton mobility. Our model can now 1) explain the behavior of perovskite‐type proton conduction in materials, and 2) predict where the material with the lowest activation energy for proton transport will be found. For materials beyond the structure tolerance limit, the temperature of phase transition is basically defined by the height of the saddle point, which in turn is inversely proportional to the second derivative of PES at zero. Thus, for a given temperature we can draw the horizontal line on a graph in Figure 4 which corresponds to the barrier height. The point, where this line crosses the phonon energy line would correspond to the composition (B to A radius ratio *r_B_/r_A_*) with the lowest activation energy.

With this insight, we can expand on the previous findings about the role of O─H bonds and unit cell volume^[^ ^21^, ^83^, ^84^ ^]^ and generalize that the specific phonon modes ‐ as shown in Figure 4 ‐ are the major predictor for the activation energies for proton conduction. For example, our predictor fully describes the behavior of barium cerate, where change of the activation energy is accompanied by dramatic change of phonon structure, and not by the change of the lattice volume. Also, it correctly explains and describes the behavior of conductivity behavior under applied strain.^[^ ^30^ ^]^

In the context of transition state theory, this phenomenon originates from significantly higher frequency of hydrogen vibration, compared to the vibrations of the lattice. That leads to compression of the horizontal isoinertial coordinate in Figure 3 and large deviation between red and green pathways. The perfect agreement of measured activation energy for proton conductivity and vibration‐related properties suggests that the amplitude of vibrations is high enough to be sensible to the protons.

**Figure 7:**
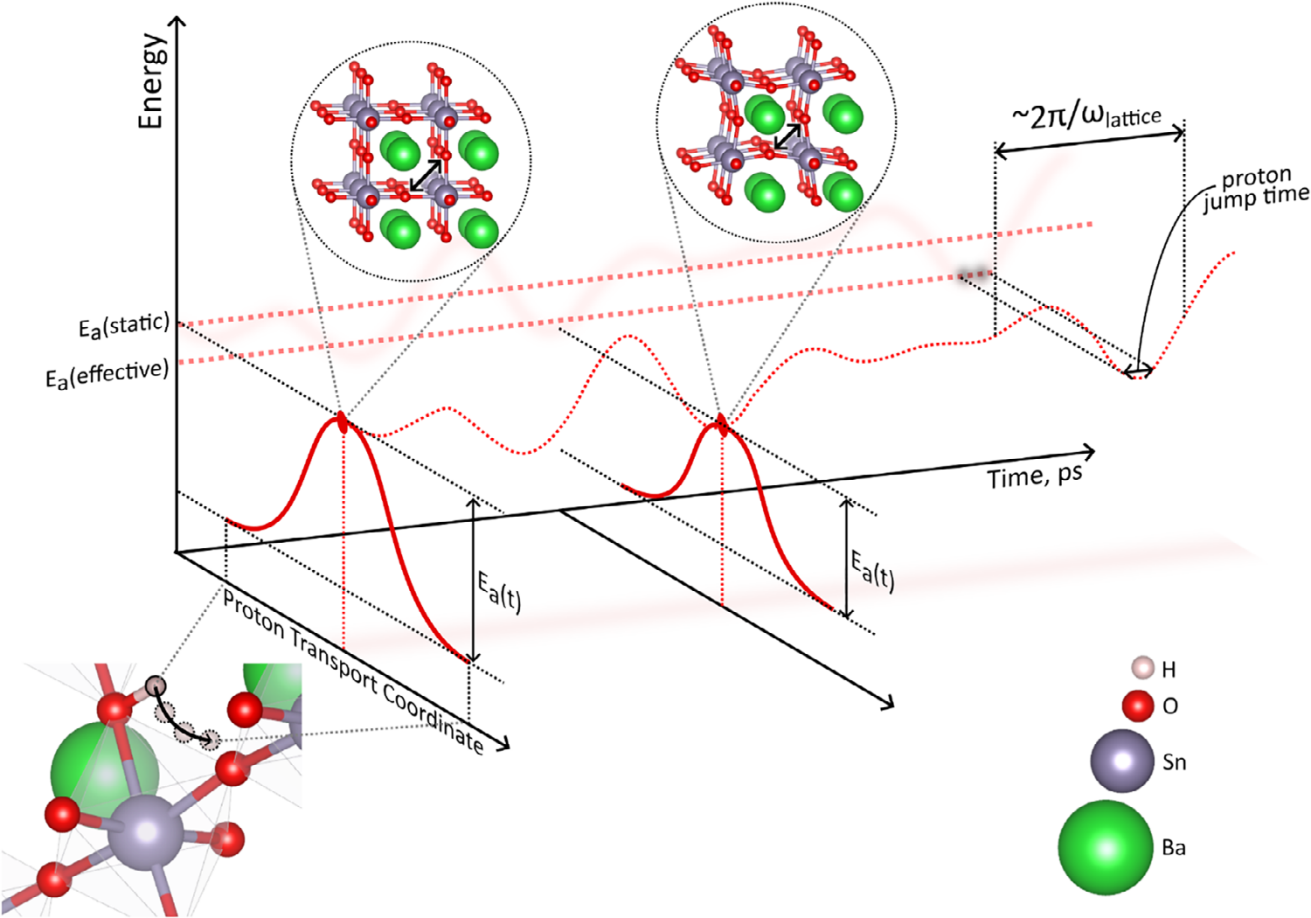
Schematic of the relation between lattice mobility and ion mobility. E_a_(static) indicates the static activation energy that is equal to the barrier calculated for the static structure. E_a_(effective) corresponds to the temporal value of the activation energy during the ion jump.

The proposed framework can be illustrated as jumping of a proton over a dynamically oscillating barrier. Let's consider the system with the potential energy surface similar to the one illustrated in Figure 3. For any position of the system in 3(N+1)‐dimensional space we can draw a line, parallel to the horizontal axis, that would correspond to jump of a proton between adjacent oxygen atoms (out of plane axis in **Figure** **7**) and plot the potential energy (vertical axis) along that line. We can then find the height of the barrier relative to two local minima, that corresponds to configurations around initial and final state and plot it as a function of time (horizontal axis) as shown in Figure 7. That results in periodic fluctuation of the barrier height. For example, in case of proton conductors, this temporal change of the barrier is realized when oxygen atoms periodically approach or move away from each other, which decreases or increases the jump distance and hence the barrier for protons; how much every phonon mode influences this barrier is the projection length, shown in Figure 4.

The characteristic frequency of the oxygen related phonons is around 10 THz, as seen from the calculated PVDOS (Figure 5). Now, the unique feature of protons comes into action: proton are extremely light and mobile; typical O─H bond stretch vibration frequency is about 100 THz,^[^ ^58^ ^]^ one order of magnitude higher than the frequency of lattice vibrations. This means that the proton moves significantly faster than the lattice and the system can perform multiple jump attempts along the proton movement axis, and overcome the barrier when it is lower, as illustrated in Figure 7.

Thus, our findings align with the idea that the phonons have strong influence on the solid‐state conductivity. However, in contrast to the majority of works that study vibrations of the cations in case of cationic conductivity and vice versa (i.e., vibrations of the conducted ion),^[^ ^85^ ^]^ here we highlight the influence of the “immobile” sublattice on the transport of mobile ions. In other words, the oxygen sublattice phonons create dynamic potential energy surface for ion transport, as illustrated in Figure 7.

A further important aspect is the jump attempt frequency, which is associated with the vibrations of the related ion. It allows the proton to attempt a jump exactly at the very moment, or at least in a favorable narrow time window of the lowest activation energy. Therefore, protons overall would have lower attempt frequency than predicted by the vibrations of O─H bond, but at the same time lower transport barriers, and the measured activation energy of proton conductivity would be beneficently lower (effective E_a_ in Figure 7). In the two materials, considered in this work, the Y‐substituted material has slightly larger “static” E_a_ (green trajectories in Figure 3), but also larger amplitude of vibrations, so the proton can perform a jump while E_a_ is temporally lower, that results in the lower measured E_a_.

This effect should obviously not be limited to the case of protons. When the mass of the mobile ion increases, the isoinertial horizontal coordinate from Figure 3 stretches, bringing two different pathways closer to each other. In the framework of Figure 7, it means that the jump timescale becomes larger, not allowing the system to “exploit” reduced barrier. We therefore anticipate that the effect, which we introduce in this paper for the proton, could decrease for other elements and eventually fade away along the periodic table, where traditional variations of transition state theory can be applied. Yet, some evidence hints that lithium, the second lightest ion, may be following^[^ ^86^ ^]^ this new transport theory.

## 4 Conclusions

We have demonstrated how nuclear resonance vibration spectroscopy can be used to establish correlations between the vibration properties and the proton conductivity activation energy. Based on experimental observations, we have shown that lattice dynamics plays the dominant role in defining the activation energy for proton transfer. We proposed a transport model that can predict the activation energy based on the structure of the material, where the main predictor is the vibration properties, and not the static lattice. Thus, we expand the previously proposed idea that lattice dynamics is indeed important in proton transport and show in detail which properties of the material exactly define the vibration properties. The substitution with larger ion, as probed with NRVS, causes phonon softening which considerably lowers the activation energy for proton transport. We have demonstrated not only the causal relation between phonon frequencies and proton conductivity but also shown causal relationship between the structure of material, vibration properties and proton jump activation energy. Then we could take a step further to expand the understanding of ionic conductivity in solid state in general, including the important influence of lattice vibrations.

## 5 Experimental Section

### Synthesis of BaSnO_3_ and BaSn_0.9_Y_0.1_O_3_

Precursor materials were BaCO_3_ (CAS 513‐77‐9), Y_2_O_3_ (CAS 1314‐36‐9), HNO_3_ (CAS 7697‐37‐2, 70%, purified by redistillation), all from Sigma–Aldrich with trace metal basis purity ≥99.999%.

Tin ^119^Sn metal foil, according to specification enriched with ^119^Sn to 96.3%, was purchased from NEONEST AB in Sweden (www.buyisotopes.com, concentration there confirmed by mass spectrometry). Tin metal sheets of 1 mm thickness with natural distribution of isotopes were purchased from Goodfellow, (SN000441 Tin, 99999% purity).

The tin metals were each dissolved in heated nitric acid for 1 h. The white precipitates were left overnight, then diluted with distilled water (produced on‐site with MicroPure Thermo Fisher Scientific, 18.2 MΩ · cm), then filtered on a paper filter and further washed until pH neutral. The resulting powders, SnO_2_ · x H_2_O, and ^119^SnO_2_ · x H_2_O, respectively, were then calcined in air at 1373 K, which was accompanied by weight loss resulting in SnO_2_ and ^119^SnO_2_ powders, respectively. Yield at this stage was 99.5%.

Then, stoichiometric amounts of SnO_2_, BaCO_3_ and Y_2_O_3_ were mixed, ground in agate mortar and then calcined at 1723 K for 2 h. The obtained reaction product was collected from the crucibles, ground again and compacted to pellets with 1 mm thickness and 9 mm diameter at a pressure of 150 kN. Those were sintered for 2 h at 1873 K. For neutron diffraction measurements, the powder samples were calcined at the same conditions without pressing.

Resulting pellets were dried in a tube furnace at 723 K under flow of dry nitrogen overnight, then dry samples were sealed and stored in a laminated foil pouch bag.

### X‐Ray Diffraction

Diagnostic crystallographic phase analysis was carried out with powder X‐ray diffraction using a PANalytical X'Pert Pro MPD diffractometer with Cu K_α_ radiation. The diffraction patterns suggested single phase BaSnO_3_ and BaSn_0.9_Y_0.1_O_3_. The X‐ray diffraction patterns are presented in Figure S1 (Supporting Information).

### Neutron Diffraction

For neutron diffraction, powder samples were used, prepared as described above from natural‐abundance isotope tin foil from Goodfellow. Neutron powder diffractograms were recorded at the HRPT neutron beamline at the Swiss Spallation Neutron Source in Villigen, Switzerland.^[^ ^87^, ^88^, ^89^ ^]^ Diffractograms were acquired at neutron wavelength 1.1545 Å at temperatures of 1, 100, and 200 K. The samples were conditioned by annealing at 400 °C under flow of dry nitrogen (“dry” sample), dry nitrogen bubbling through water or D2O to achieve the dry and water‐saturated states. The samples were transferred and measured in sealed containers with minimal exposure to atmosphere. The stoichiometry as determined by Rietveld refinement of the neutron diffraction data is in Table S1 (Supporting Information). Sn occupancy is 0.970±0.003 and O occupancy is 0.975±0.003, so given the nominal stoichiometry BaSnO_3_, it should be BaSn_0.97_O_2.925_. Hence, ≈1.5% of Sn are Sn^2+^: net charge = 0 = 2.925*(‐2) + 1 · (+2) + (0.97‐x) · (+4) + x· (+2); x = 0.015. The nominal BaSnO_3_ contains thus a concentration of oxygen vacancies. Therefore, there exist vacancies allowing for proton transport even without substituting the Sn^4+^ by Y^3+^.

### Impedance Spectroscopy

For impedance measurements, the pellets were metallized with platinum paste (Metalor Technologies SA) on top and bottom sides and fired at 1223 K and then contacted with platinum mesh. Measurements were performed in a ProboStat® (Norecs, Trondheim, Norway) at temperatures ranging from 473 to 973 K under three controlled atmospheres, provided by a HumiStat (Norecs, Trondheim, Norway): flow of dry nitrogen, and under nitrogen saturated with H_2_O water vapor, and with D_2_O heavy water (Sigma‐Aldrich, 99.9% D2O) vapor. Samples were kept in the corresponding atmosphere at 523 K for at least 24 h prior to the measurements.

With the molar mass of BSY10 of 569.31 g mol^−1^ and that of water be 9.01 g mol^−1^, it determined the proton concentration of the dry (mass m_dry_) and humidified (mass m_wet_) samples gravimetrically^[^ ^90^ ^]^ with a microbalance to

(7) $$\begin{array}{ccc} \left[OH^{\cdot }\right] & = & \left[H^{+}\right]\\ & = & \frac{m_{wet}-m_{dry}}{m_{dry}}\cdot \frac{M_{BSY10}}{0.5\cdot M_{H2O}}=\frac{604.11mg-603.72mg}{603.72mg}\\ & & \cdot \frac{569.31}{9.01}\approx 0.01977\cdot 63.186\approx 1.25\;mol\% \end{array}$$

Impedance spectra were recorded using a Solartron 1260 gain phase analyzer, equipped with an impedance range extender (Solartron Dielectric Interface Model 1296 A). The scanned frequencies ranged from 1 MHz to 0.1 Hz, with the amplitude of the potential set to 100 mV. Quantitative analysis of the impedance spectra using the equivalent circuit method was carried out with Zmeam makro,^[^ ^91^ ^]^ which runs on Igor Pro (Wavemetrics Inc.).^[^ ^92^ ^]^ Attribution to various pathways (bulk and grain boundary conductivity) and charge carriers was done in accordance to previously published data^[^ ^93^, ^94^ ^]^ and to the difference between measurements with normal and heavy water. The raw impedance spectra and equivalent circuit are shown in Figures S8 and S9 (Supporting Information).

### Nuclear Resonant Vibration Spectroscopy

Nuclear resonant vibration spectra (NRVS) measurements were collected at beamline BL35XU at SPring‐8 in Hyogo, Japan.^[^ ^95^, ^96^ ^]^ The samples were dried by the same procedure as for the impedance measurement and transferred to the beamline in a sealed pouch bag, prior to measurement. NRVS spectra were then processed using the online “NRVS Tool” from spectra.tools^[^ ^97^ ^]^ based on software package of PHOENIX.^[^ ^98^ ^]^ Excitation energy was 23.87 keV. Acquisition of one spectrum covering the range from ‐30 to 120 meV took ≈24 h. All the measurements were performed at ambient temperature under vacuum.

### Atomistic Simulations of Vibrational Properties

Simulation of element‐projected vibrational density of states was performed by combination of density functional theory and molecular dynamics with fine‐tuned universal atomistic machine‐learning interatomic potential (MLIP) MatterSim version 1.2.0.^[^ ^99^ ^]^ Density functional theory (DFT) calculations were performed with the Quantum ESPRESSO package^[^ ^100^, ^101^, ^102^ ^]^ with generalized gradient approximation (GGA) as parametrized by Perdew, Burke and Ernzerhof (PBE functional)^[^ ^103^ ^]^ with Hubbard U correction. Core electrons were treated with projector augmented wave pseudopotentials available in the standard solid‐state pseudopotentials (SSSP) library (http://materialscloud.org/sssp).^[^ ^104^ ^]^ The Hubbard U term was applied to O 2p electrons with ortho‐atomic Hubbard projectors, which is common approach for Sn^4+^ compounds.^[^ ^105^ ^]^ In all calculations, the cutoff energy was 80 Ry for the kinetic energy and 600 Ry for the charge density. Brillouin zone integration was performed with Gaussian spreading with 0.01 Ry.

First, the Hubard U parameter for DFT calculations was optimized by matching the simulated Sn‐projected PVDOS to the NRVS‐derived PVDOS of pure BaSnO_3_. Phonon structures were calculated with the finite displacement method using the PHONOPY package.^[^ ^106^, ^107^ ^]^ Calculations were done with the same parameters using an 8x8x8 Monkhorst‐Pack shifted grid of k‐points. Force constants were calculated using a 3x3x3 supercell. Brillouin zone integration was performed on a 20x20x20 mesh. Optimized value of Hubbard U parameter was 7 eV, which is also validated by a perfect match with the experimental bandgap (see Figure S11, Supporting Information and reference by Aggoune et al.^[^ ^108^ ^]^).

To simulate the phonon structure of the doped and defect‐containing systems, a finetune of the pre‐trained model MatterSim‐v1.0.0–5 M was performed, available in MatterSim repository. For the finetune, the single‐point DFT calculations of total energy and forces were performed for the various randomized configurations of BaSnO3 supercells with random atom displacements, oxygen vacancies and yttrium substitution (maximum supercell size 3 × 3 × 3). The finetune was performed with the training script, included in the MatterSim package. Cutoff radius was set to 5.0, three‐body cutoff to 4.0, energies and forces were included, and stresses not included. Further details of the training set generation, the full training set, descriptive statistics of the training set, and the finetuned model along with its evaluation on the test set are available in Comparison of the phonon properties of BaSnO_3_ and BaSn_7/8_Y_1/8_O_3_ pre‐trained model, finetuned model and DFT are shown in Figures S4 and S5 (Supporting Information). The mean average errors for the test set, that is similar to the configurations, used in the manuscript, were 7.4 meV for energies and 37.4 meV/Å for forces.

The vibrational properties of the experimentally studied materials were simulated with the finetuned MLIP using 3x3x3 supercells with random defects (Y substitution and O vacancies). For BaSnO_3_ it created 0‐6 oxygen vacancies, for BSY, 3 random Sn atoms were replaced with Y atoms, and 0‐6 oxygen vacancies were generated. For each system the geometry was optimized, then force constants were calculated by finite displacements method using this 3 × 3 × 3 supercell with the displacement amplitude 0.01 Å, and phonon modes were integrated over Brillouin zone using 8 × 8 × 8 mesh. All phonon calculations were performed using PHONOPY package. The configurations that had imaginary phonon frequencies were discarded. Total of 30 configurations for each composition were simulated this way, except for the non‐doped configurations with 0 or 1 vacancy, where only 1 configuration was calculated. For the figures presented in the manuscript, it chose the configuration with the best fit to the experimental data, which was 1 vacancy for BaSnO_3_ and 2 vacancies for 3 × 3 × 3 BaSn_1‐x_Y_x_O_3_ supercell and 1 vacancy for 2 × 2 × 2 BaSn_1‐x_Y_x_O_3_ supercell. The reported values (phonon DOS, thermal displacements etc.) were obtained by getting weighted average over all simulated systems with Boltzmann weights, calculated using the potential energy of the system. In addition, for BSY it also simulated 2 × 2 × 2 supercell with 1 Y atom and 1 random oxygen vacancy to represent the configurations with superstructure. Optimized lattice parameters were: 4.149 Å for BaSnO_3_ 3 × 3 × 3 supercell with 1 O vacancy, 4.178 Å for 3 × 3 × 3 BaSn_1‐x_Y_x_O_3_ with 2 O vacancies. Gaussian 1D filter with sigma = 0.64 meV was applied to the simulated phonon DOS for comparison with experimental data. Sigma value was derived from FWHM of the elastic peak, which was taken as instrument resolution. Comparison of different defect concentrations and experimental DOS are given in Figure S6 (Supporting Information). For visualization of phonon modes, the web‐based tool was used (https://henriquemiranda.github.io/phononwebsite/). Phonon dispersion curves were calculated on simple systems (BaSnO3 unit cell, 2 × 2 × 2 supercell with 1 Y atom) for clarity of presentation. The phonon dispersion curves for BaSn_7/8_Y_1/8_O_3_ were calculated for a 2 × 2 × 2 supercell and projected onto the primitive cell of BaSnO_3_ using the phonon band unfolding method.^[^ ^109^ ^]^ The phonon dispersion curves for BaSnO_3_ were plotted with the same tool for consistency and ease of comparison. Both plots were produced with Lorentzian smearing with gamma = 0.05 THz.

The proton transport pathways were calculated using the climbing nudged elastic bands (NEB) method, which is implemented in the Quantum Espresso package. For these calculations, it used a 2 × 2 × 2 super‐cell of BaSnO_3_ and BaSn_7/8_Y_1/8_O_3_, respectively. To screen for local minima, it first placed a single hydrogen atom at all possible symmetrically inequivalent positions within the cell, using a grid increment of ≈0.4 Ångstrom.

It then calculated the total energy of the system at each position to map the energy as a function of the proton's position without structure relaxation. The 3D map and isosurfaces are presented in the Supporting Information, Figure S12 (Supporting Information). Next, a hydrogen atom was placed in all possible inequivalent local minima, the geometry was re‐laxed, and subsequently, NEB calculations were performed for the path between the closest minima. For each NEB calculation, it used five intermediate images, and the image with the highest energy was allowed to climb along the path. These calculations were performed in two modes: 1) only the hydrogen atom was allowed to change position, and 2) all atoms in the simulation cell were allowed to change positions. To estimate the vibrational contributions to the barrier height, it calculated the phonon properties of the initial equilibrium state and the transition state using PHONOPY.^[^ ^106^, ^107^ ^]^

For the force constants calculation, the same unmultiplied simulation cell (2 × 2 × 2 unit cells) were used; the phonon DOS was calculated with the same parameters as for other systems of the same size. Thermodynamic properties were calculated on a 21 × 21 × 21 mesh. The difference of vibrational enthalpy at 600 K was used for the barrier height correction (Table S2, Supporting Information).

Phonon properties of the Y‐doped BaZrO_3_ and BaCeO_3_ were calculated using 2 × 2 × 2 supercells where one B‐site atom (Zr or Ce) was substituted with Y. These calculations employed the same DFT parameters as described in the previous section. For calculating the deformed structure, the displacement with a pattern 1 shown in Figure 4c was applied. The potential energy surfaces along a specific phonon mode were calculated with DFT for all considered materials using 2 × 2 × 2 supercells, a size that coincides with the periodicity of the mode at the edge of the Brillouin zone. To estimate the influence of lattice vibrations on the barrier height, it developed a novel method involving the projection of atomic displacements onto the phonon modes. For this purpose, it first defined a vector (Δ𝑋) perpendicular to the vector connecting the initial and final states (corresponding to the vertical axis in Figure 3d). This vector was calculated as follows:

(8) $$\Delta X=\left(X_{eqi}-X_{TS}\right)-\;\frac{\left(X_{eqi}-X_{TS}\right)\cdot \left(X_{eqf}-X_{eqi}\right)}{∥X_{eqf}-X_{eqi}{∥}^{2}}\left(X_{eqf}-X_{eqi}\right)$$

here, X_TS_, X_eqi_, X_eqf_ are 3N – dimensional position vectors (N is number of lattice atoms), corresponding to the transition state, initial and final equilibrium positions of the lattice atoms, respectively. Then, for each phonon mode, it calculated the modulus of the projection of its displacement vector onto the ΔX vector. To plot the band structure, it first calculated the phonon modes for theBaSnO3 unit cell along a specific path in the Brillouin zone. It then multiplied the cell to create the supercell and calculated the corresponding displacement vector by multiplying the eigenvector by the respectiveq‐vector. The thickness of each plotted line was set equal to the squared length of its projection. For the projected partial vibrational density of states (PVDOS), it used a 12 × 12 × 12 mesh in the Brillouin zone. The states were integrated over this mesh, and the squared length of the projection was plotted against the wavenumber of the corresponding mode. A Gaussian 1D filter with σ ≈ 3 cm^−1^ was applied to the resulting data. The code that implements the calculations is available at https://github.com/alexey‐rulev/plus_vibe.

### Statistical Analysis

The data originate from deterministic computational calculations (DFT, MLIP‐based molecular dynamics, PHONOPY finite‐displacement phonon calculations, NEB calculations) and from single‐run experimental measurements (neutron diffraction, impedance spectroscopy, nuclear resonant vibration spectroscopy). As these methods generate fully deterministic output rather than stochastic samples, no inferential statistical hypothesis testing is applicable. Statistical procedures therefore are of descriptive processing steps appropriate to the respective data modality, as detailed below.

### Pre‐Processing of Data

No mathematical transformation, normalization, or outlier removal was applied to DFT, PHONOPY, NEB, or MLIP‐generated datasets, as these workflows are intrinsically deterministic and contain no random noise. For the MLIP training and test sets, descriptive statistics—including distributions of forces, energies, and interatomic distances—are provided in the Zenodo repository.^[^ ^110^ ^]^ For experimental datasets (impedance spectroscopy, NRVS, neutron diffraction), raw spectra are baseline‐corrected using instrument‐standard routines; no statistical exclusion of data points was performed.

### Data Presentation

All reported values from DFT, MLIP, NEB, and phonon calculations represent deterministic results from fully converged simulations. For MLIP evaluation, mean absolute error (MAE) metrics are reported for energies (meV/atom) and forces (meV/Å). For impedance data, conductivity values are shown as fitted results from deterministic equivalent‐circuit modeling. For NRVS data, the PVDOS was obtained using PHOENIX‐based processing.^[^ ^98^ ^]^ Where distributions (e.g., phonon DOS, force distributions) are shown, they represent configurational sampling, not stochastic variability.

### Sample Size (n)

For MLIP training and testing, n is the number of atomic configurations in each subset as listed in the Zenodo record.^[^ ^110^ ^]^ For phonon simulations, n denotes the number of independently generated defect configurations per composition (typically 30; for BaSnO_3_ with 0–1 vacancy only one configuration was used). For NEB calculations, n denotes the number of images (five intermediate images per pathway). For experimental data, n corresponds to a single measurement per sample under each specified condition (no statistical averaging across multiple samples was required).

### Statistical Methods

Because the work does not involve stochastic sampling or repeated biological/chemical measurements, no statistical hypothesis testing, P‐values, alpha values, or post‐hoc adjustments is used. Descriptive metrics (MAE, RMS error, averages over configurational ensembles, Boltzmann‐weighted averages, or Gaussian convolution parameters) are reported where relevant. All distributions shown (e.g., phonon DOS, force distributions, projected density of states) reflect deterministic aggregation over simulated configurations and not sampling variability.

### Software

Statistical and computational analyses were performed using: Quantum ESPRESSO (DFT, NEB), PHONOPY (finite‐displacement phonons), MatterSim 1.2.0 (MLIP fine‐tuning), Igor Pro + Zmeam macro (impedance spectroscopy fits), PHOENIX / spectra.tools (NRVS processing), as described in the Methods Section.

## Conflict of Interest

The authors declare no conflict of interest.

## Supporting information



Supporting Information

Supporting Video

## Data Availability

The data that support the findings of this study are available from the corresponding author upon reasonable request.
