# Supplementary material for: Strong Proton‐Phonon Coupling Drives Fast Ion Transport in Perovskites
Source: Adv Sci (Weinh). 2025 Dec 12;13(9):e07261. doi: 10.1002/advs.202507261 (PMC12904003; doi:10.1002/advs.202507261)
Supplement: Supplementary file 1 — Supporting Information [file ADVS-13-e07261-s001.docx]

Supporting Information

Strong Proton-Phonon Coupling Drives Fast Ion Transport in Perovskites

*^1^Alexey Rulev^*^, ^2^Nobumoto Nagasawa, ^3^Hongxin Wang, ^4^Vladimir Pomjakushin, ^5^Martin Kunz ^2^Yoshitaka Yoda, ^3^Stephen P. Cramer, ^6^Qianli Chen, ^1^Artur Braun^*^*

**Table S1**. Structure parameter from Rietveld refinement for the BaSnO_3_ sample and BaSn_0.9_Y_0.1_O_3_ sample.

| BaSnO_3_, wR = 5.11% | | | |
| --- | --- | --- | --- |
| T, K | 1 K | 100 K | 200 K |
| a, Å | 4.11016(7) | 4.11113(10) | 4.11329(11) |
| Ba occ. | 1 | | |
| Sn occ. | 0.970(3) | | |
| O occ. | 0.975(3) | | |
| U_iso_(Ba), Å^2^ | 0.00167(12) | 0.00272(17) | 0.00402(18) |
| U_iso_(Sn), Å^2^ | 0.00068(10) | 0.00120(15) | 0.00184(16) |
| U_11_(O), Å^2^ | 0.00498(11) | 0.00571(17) | 0.00768(19) |
| U_33_(O), Å^2^ | 0.00228(21) | 0.00299(31) | 0.0033(3) |

| BaSn_0.9_Y_0.1_O_3_, wR = 6.36% | | | | | | |
| --- | --- | --- | --- | --- | --- | --- |
|  | BaSnO_3_ | | | BaSn_1-x_Y_x_O_3_ | | |
| Weight fr. | 0.211(9) | | | 0.789(9) | | |
| Ba occ. | 1 | | | 1 | | |
| Sn occ. | 0.964(18) | | | 0.854(4) | | |
| Y occ. | — | | | 0.1337(23) | | |
| O occ. | 0.999(18) | | | 0.952(6) | | |
| T, K | 1 K | 100 K | 200 K | 1 K | 100 K | 200 K |
| a | 4.10981(20) | 4.11109(20) | 4.11335(22) | 4.14138(19) | 4.14269(19) | 4.14533(21) |
| U_iso_(Ba), Å^2^ | 0.0000(7) | 0.0008(7) | 0.0010(7) | 0.00392(27) | 0.00478(27) | 0.00611(30) |
| U_iso_(Sn), Å^2^ | 0.00015(6) | 0.00013(6) | 0.0024(6) | 0.000(11) | 0.00283(17) | 0.0064(13) |
| U_iso_(Y), Å^2^ | — | | | 0.02(5) | 0.0032(8) | 0.008(3) |
| U_11_(O), Å^2^ | 0.0036(7) | 0.0053(7) | 0.0060(7) | 0.00882(30) | 0.00965(31) | 0.0110(3) |
| U_33_(O), Å^2^ | 0.0002(11) | 0.0015(11) | 0.0014(11) | 0.0061(4) | 0.0061(5) | 0.0066(5) |


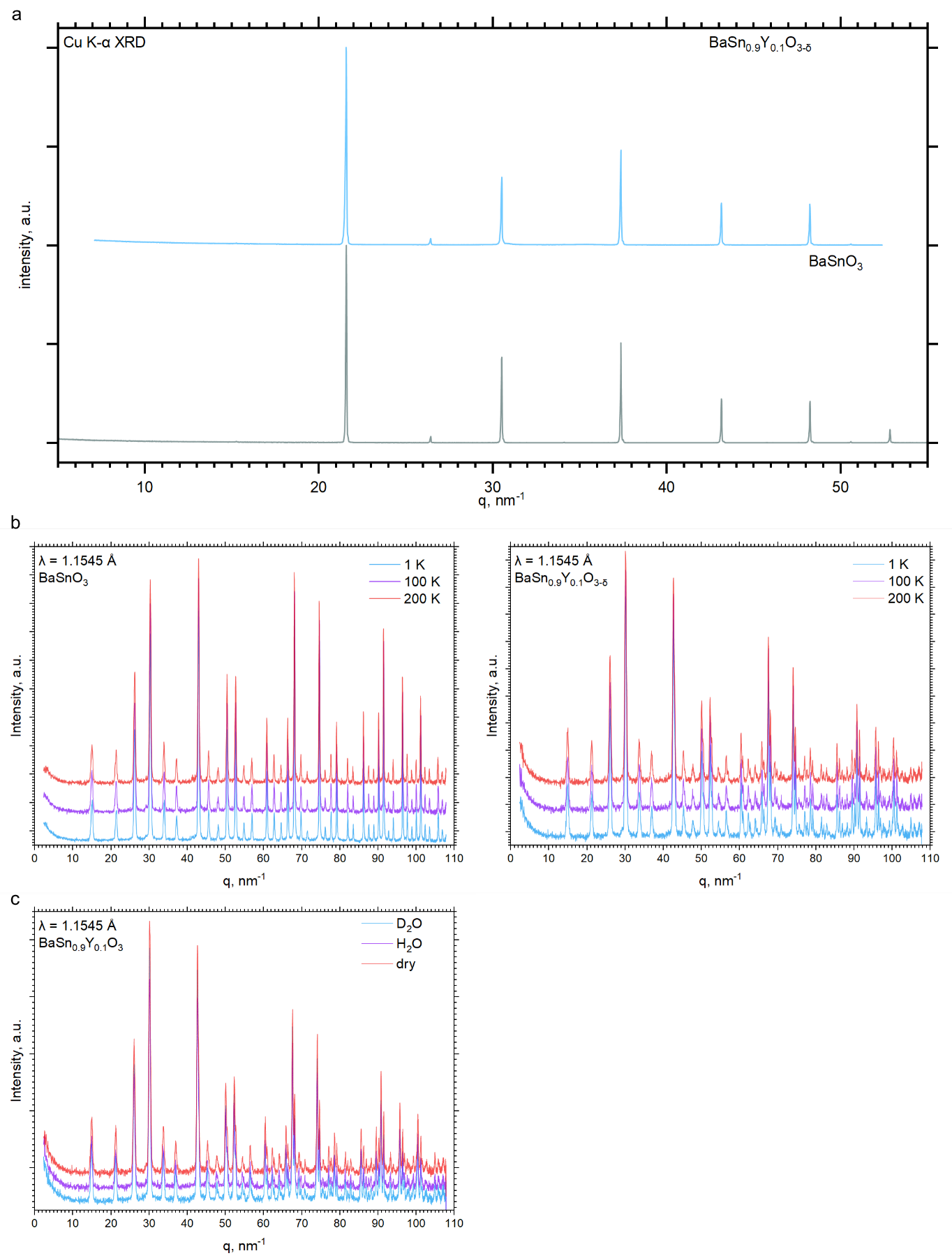


**Figure S1**. (A) X-ray diffraction patterns of BaSnO_3_ and BaSn_0.9_Y_0.1_O_3-δ_. (B) Neutron diffraction patterns of BaSnO_3_ and BaSn_0.9_Y_0.1_O_3-δ_ measured at 1, 100 and 200 K. The patterns are vertically offset. (C) Neutron diffraction patterns of BaSn_0.9_Y_0.1_O_3-δ_ conditioned at different atmospheres, measured at 1 K.


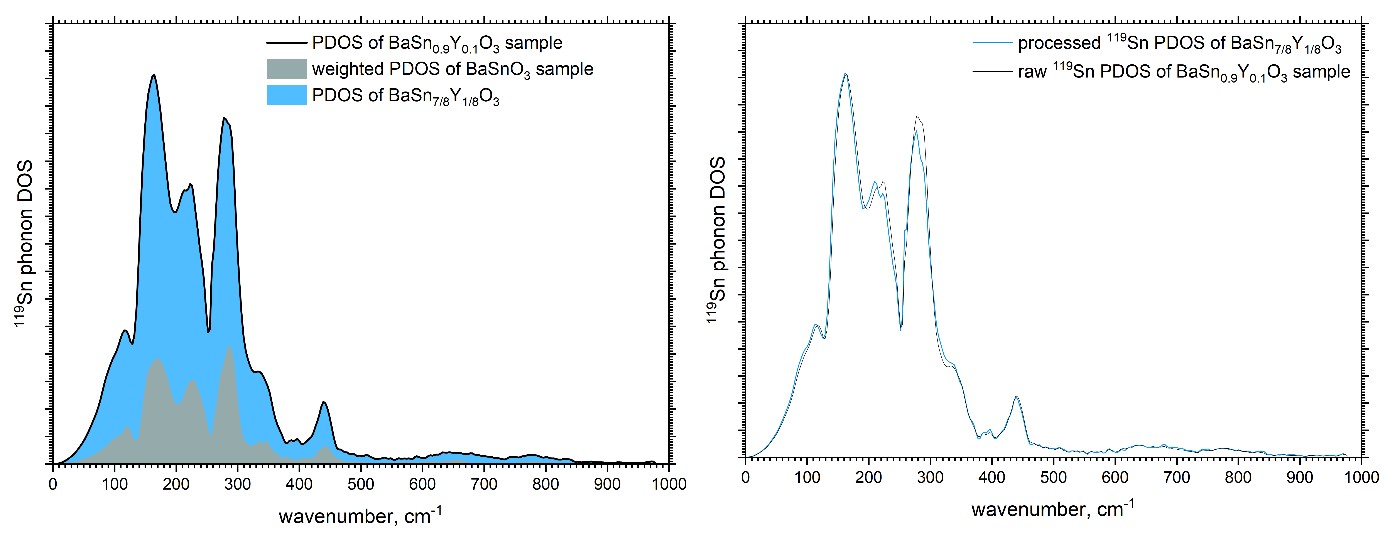


**Figure S2**. Left: illustration of weighted subtraction of BaSnO_3_ experimental PDOS from BaSn_0.9_Y_0.1_O_3-δ_ experimental PDOS to obtain PDOS of BaSn_7/8_Y_1/8_O_3-δ_. Right: comparison of measured PDOS of BaSn_0.9_Y_0.1_O_3-δ_ and processed PDOS of BaSn_7/8_Y_1/8_O_3-δ_.


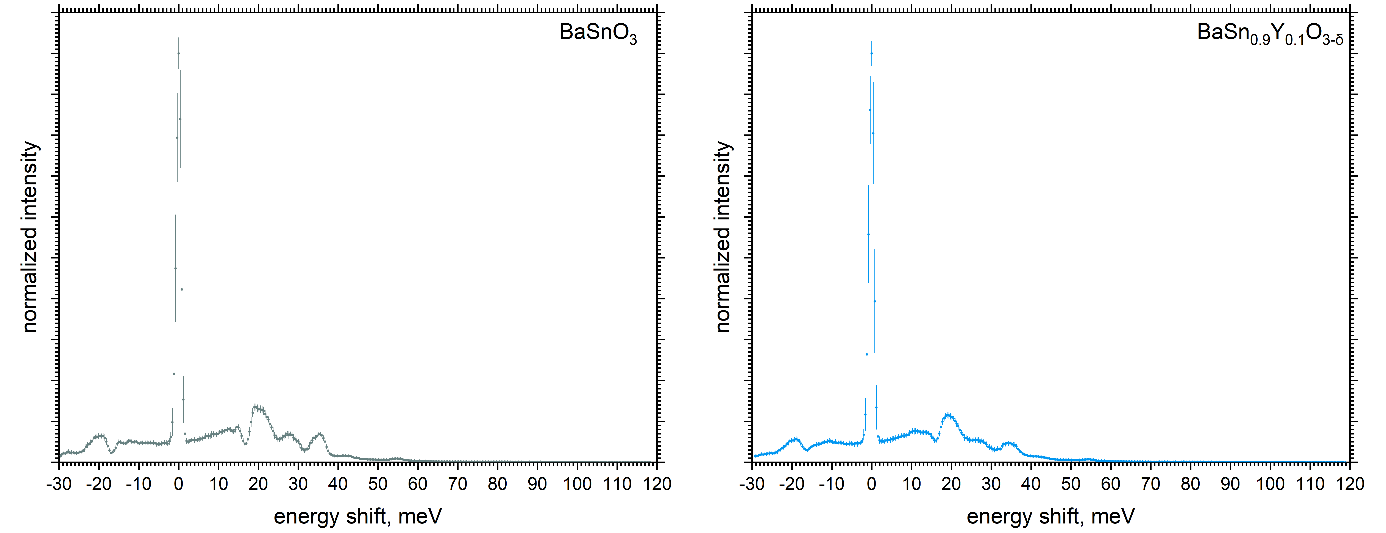


**Figure S3**. Experimental raw spectra of nuclear resonance vibrational spectroscopy of dry BaSnO_3_ (left) and BaSn_0.9_Y_0.1_O_3-δ_ (right). We have shifted the spectra so that elastic peak with the maximum intensity is at 0 energy shift.


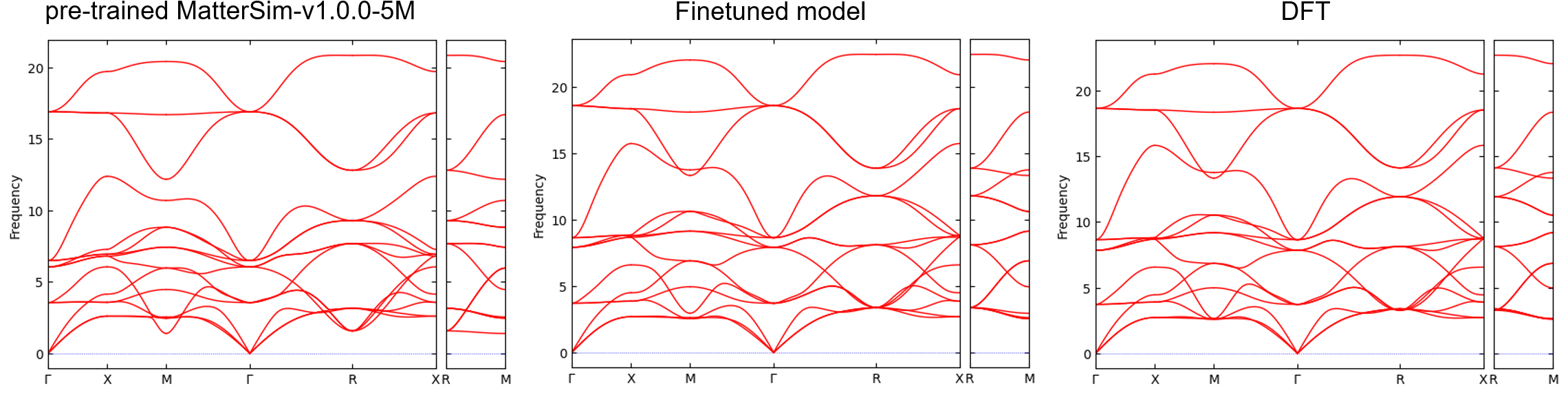


**Figure S4**. Phonon dispersion curves of BaSnO_3_ with perovskite structure, calculated with pre-trained model, the finetuned model, and DFT. Frequency is in THz.


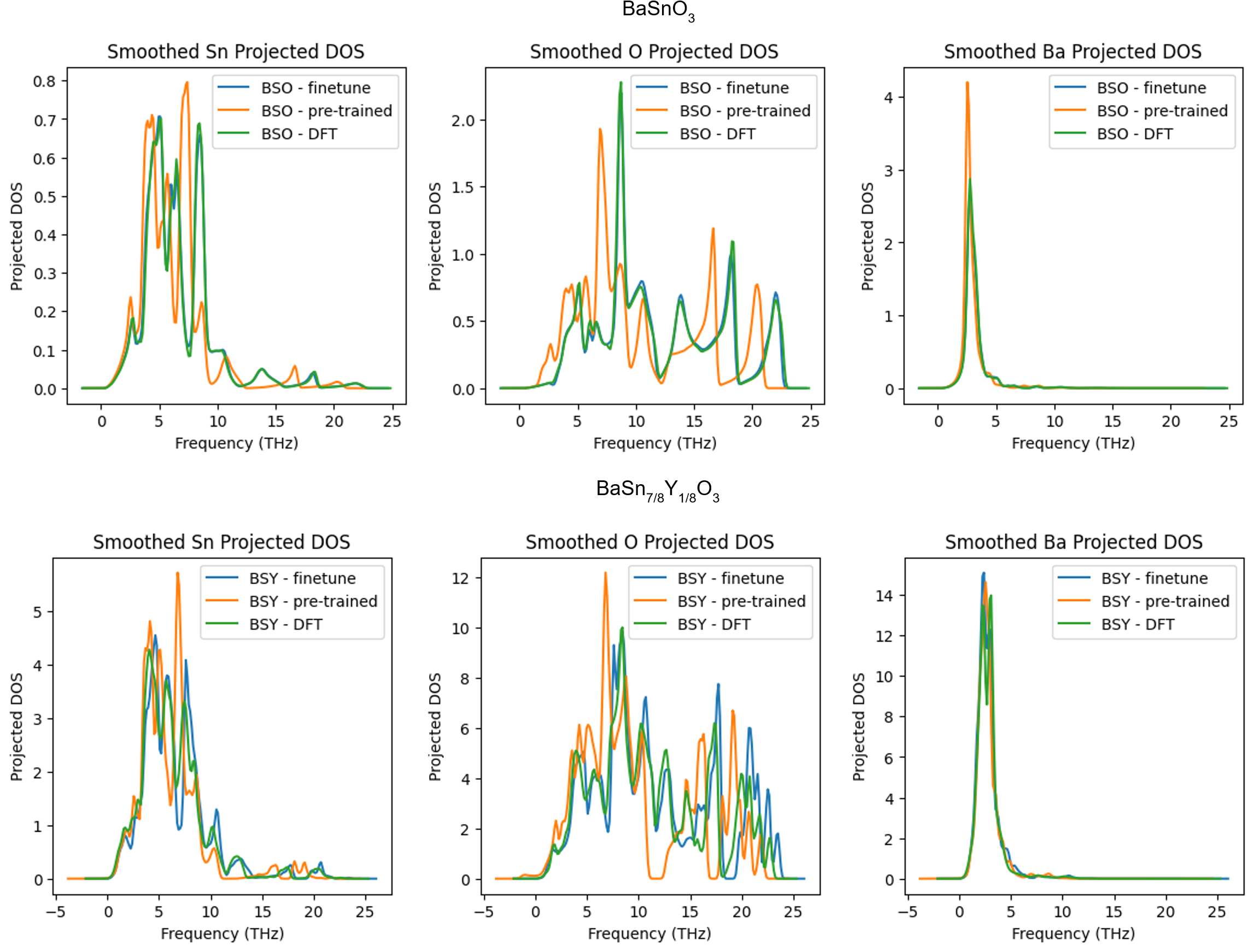


**Figure S5**. Comparison of normalized element-projected phonon DOS, calculated with pre-trained, finetuned MLIP and DFT for BaSnO_3_ and BaSn_7/8_Y_1/8_O_3_


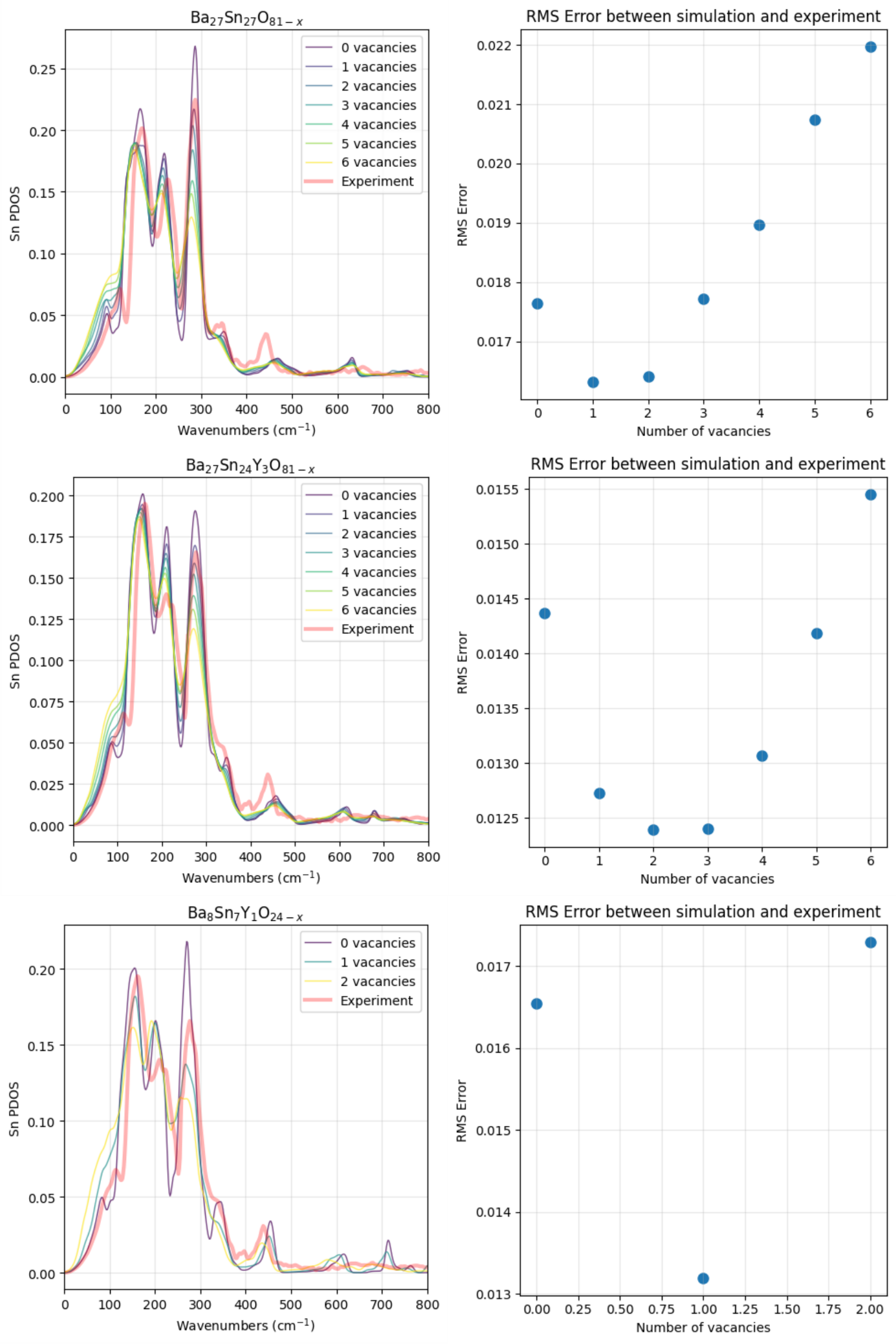


**Figure S6**. Left: comparison of simulated Sn PVDOS of BaSnO_3_ and BaSn_1-x_Y_x_O_3_ with different number of oxygen vacancies and experimental Sn PVDOS, obtained from NRVS. Right: root mean squared difference with experimental PVDOS as a function of number of oxygen vacancies.

Table S2: calculated vibrational thermodynamic parameters of the equilibrium and transition states in BaSnO_3_ and BaSn_7/8_Y_1/8_O_3_, calculated at 600 K. Values are given per simulation cell containing 1 hydrogen atom.

| Configuration (see Figure 3) | BaSnO3 | | | BaSn_7/8_Y_1/8_O_3_ | | |
| --- | --- | --- | --- | --- | --- | --- |
|  | 2 | TS 2-3 | Diff.  TS-eq. | 5 | TS 4-5 | Diff.  TS-eq |
| Free energy, eV | -3.24454 | -3.3799 | -0.13536 | -3.24687 | -3.33285 | -0.08598 |
| Entropy, eV/K | 0.016816 | 0.016843 | 2.73E-05 | 0.016814 | 0.016726 | -8.8E-05 |
| Enthalpy, eV | 6.726082 | 6.681064 | -0.04502 | 6.841802 | 6.702916 | -0.13889 |


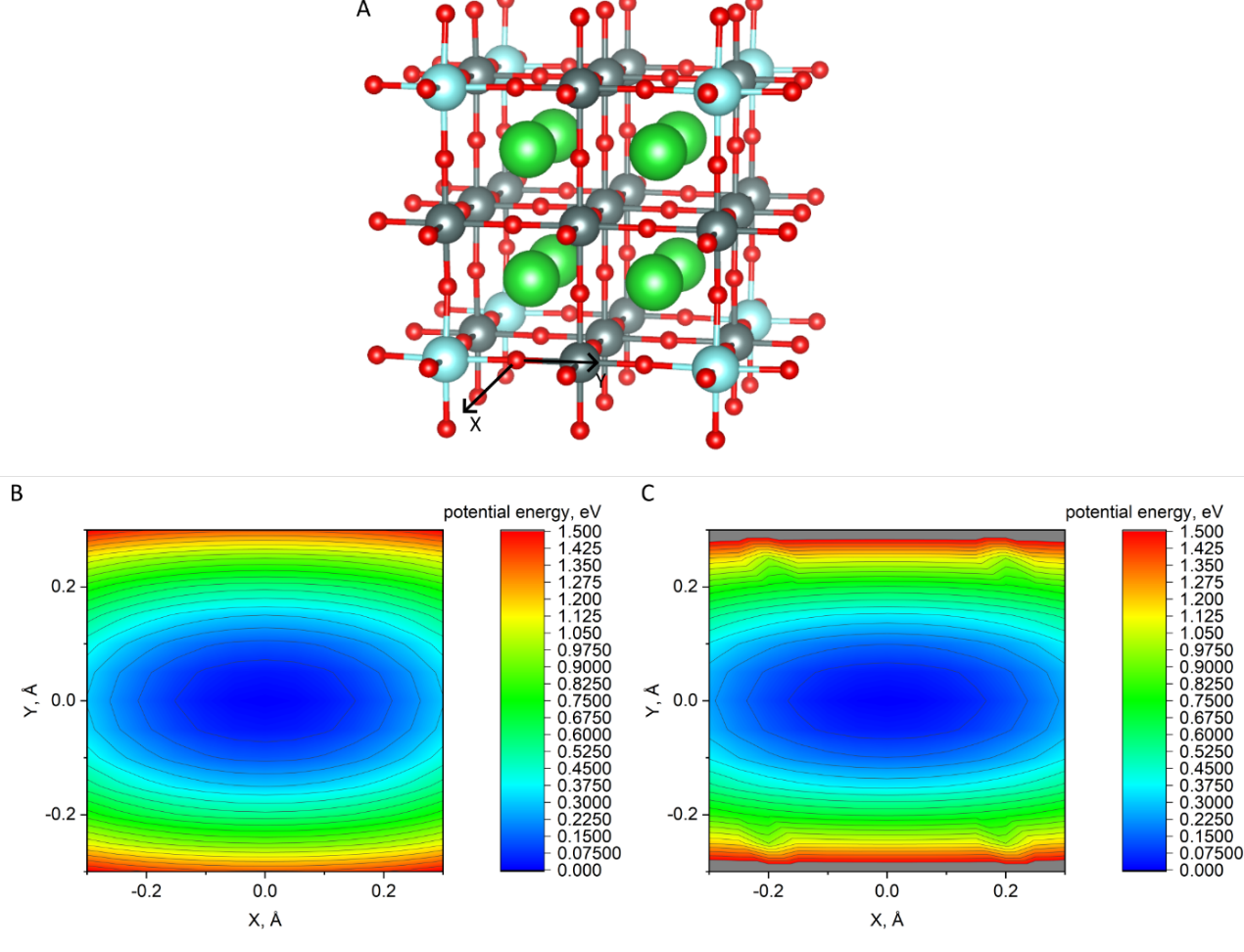


**Figure S7**. Calculated potential energy for oxygen atom displacement around equilibrium point. (A) Scheme of the structure and the directions of displacement. (B) Potential energy surface of indicated oxygen atom in BaSnO_3_ and (C) BaSn_7/8_Y_1/8_O_3_.


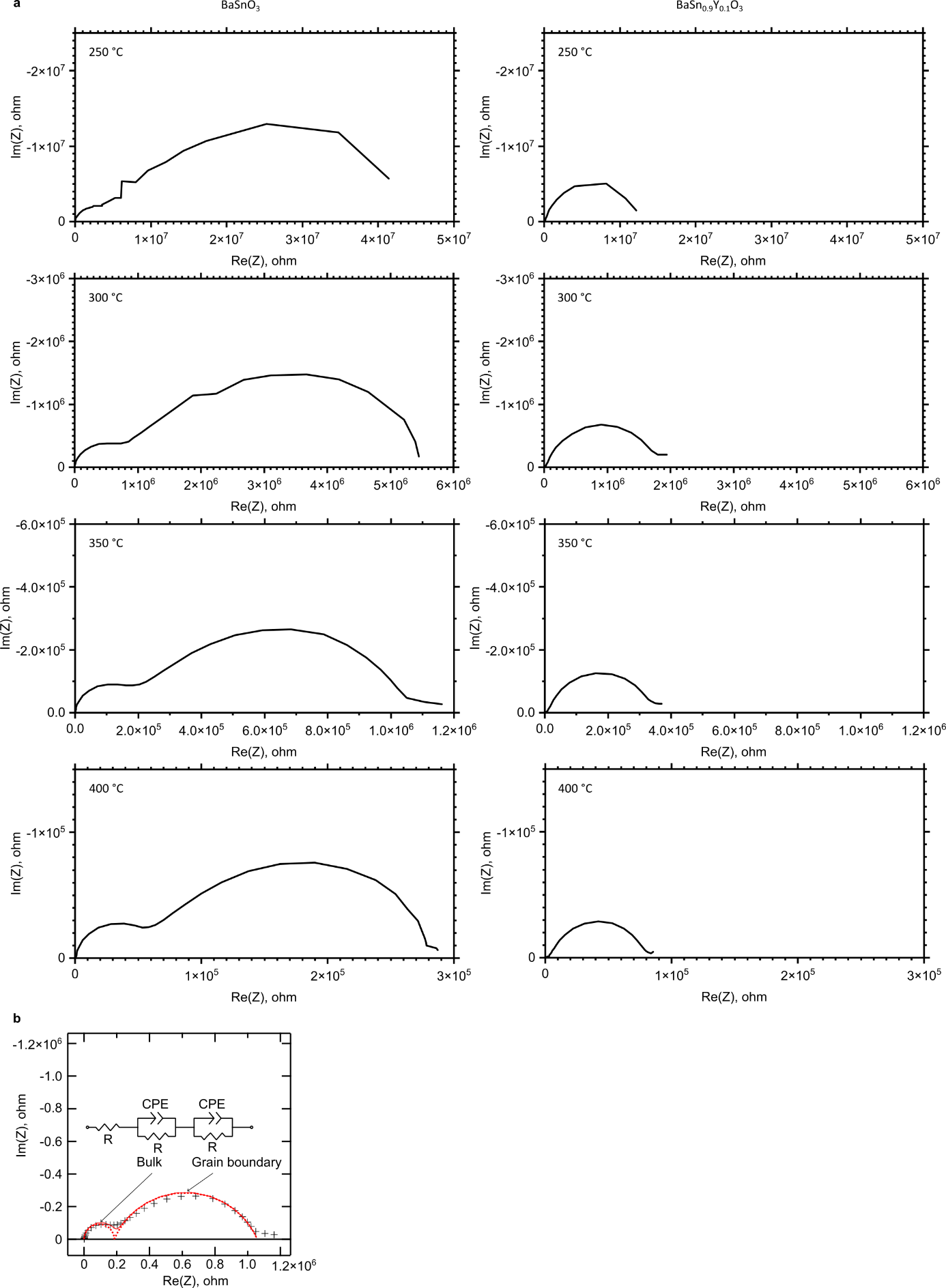


**Figure S8**. a. Raw impedance of BaSnO_3_ and BaSn_0.9_Y_0.1_O_3_ in Nyquist coordinates. b. Example of experimental spectrum and fit impedance spectrum, corresponding equivalent scheme and the element attribution.


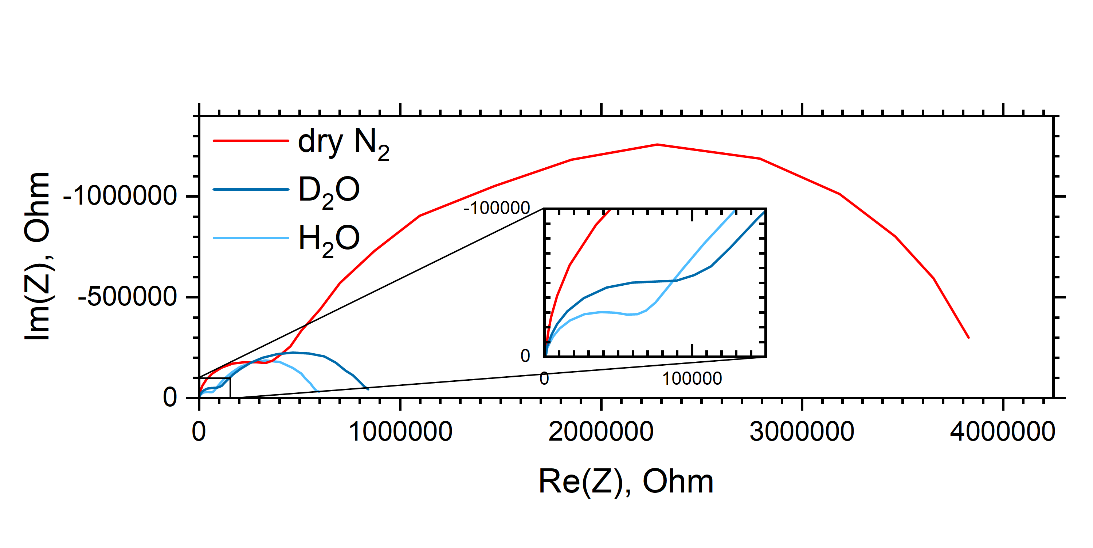


**Figure S9**. Impedance spectrum of the BaSnO_3_ pellet at 400 °C in dry N_2_, N_2_ with H_2_O and N_2_ with D_2_O vapor.


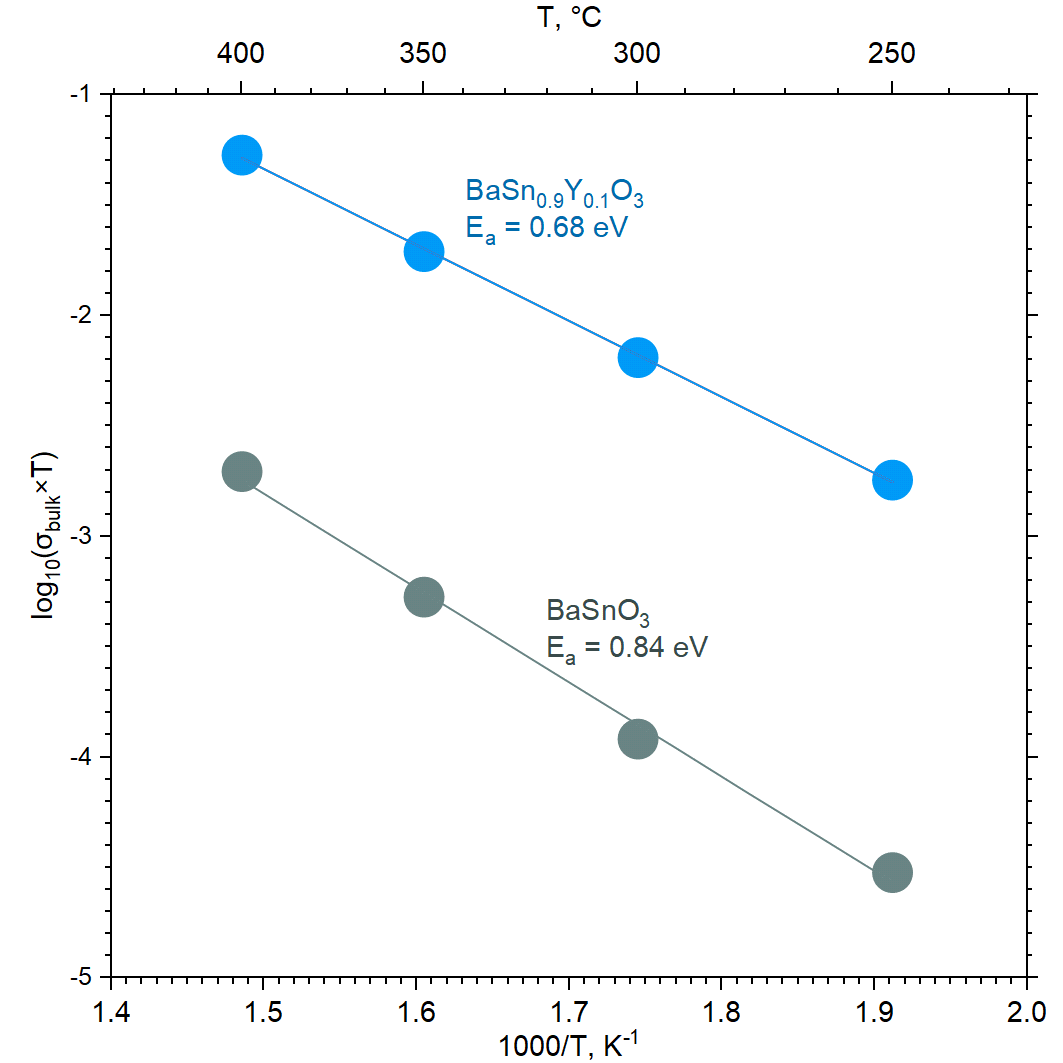


**Figure S10.** Bulk conductivity in Arrhenius coordinates measured from two samples in humid atmosphere. Solid lines are least square fits to Arrhenius' equation, the slope of which yields the activation energy. Corresponding impedance spectra and equivalent circuits are given in Figure S8.


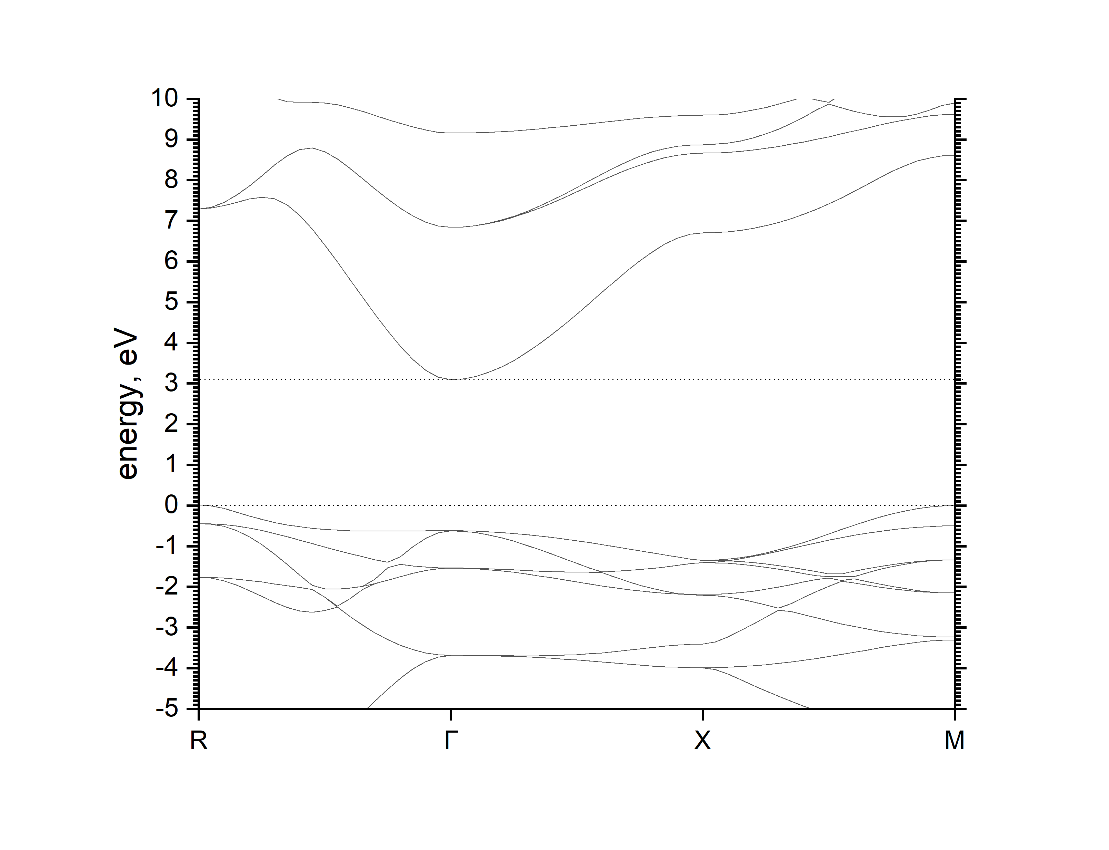


**Figure S11**. Calculated electronic band structure of BaSnO_3_. Top of the valence band is set to zero.


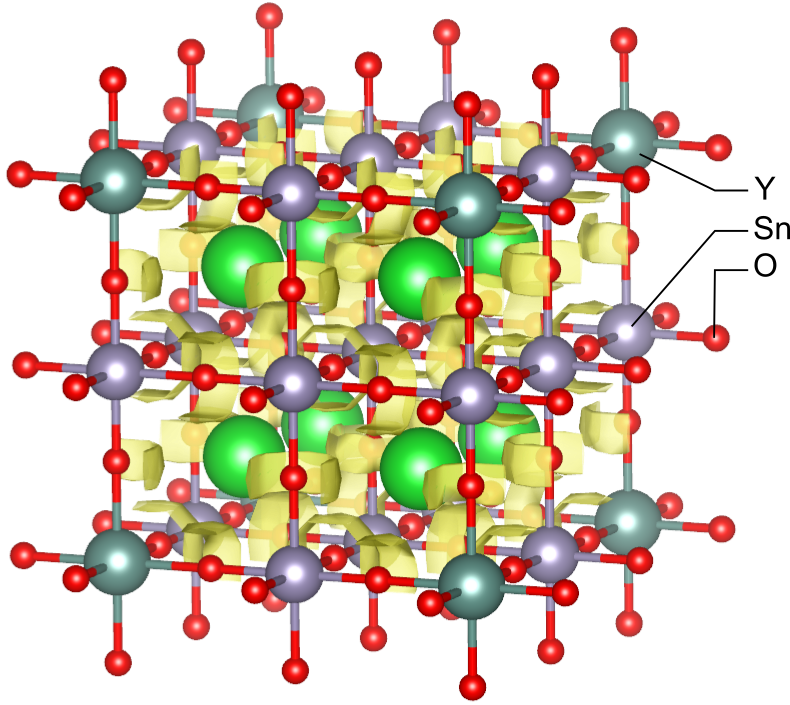


**Figure S12**. Structure of BaSn_7/8_Y_1/8_O_3_ and calculated isosurfaces, showing positions where protons have energy < 0.5 eV above the global minimum.

**Discussion**

Arrangement of Y atoms in BSY.

To experimentally check for the existence of a superstructure, we carried out anomalous XRD, which provides x-ray contrast variation [87]. The XRD patterns were recorded at energies around the yttrium K-edge (Figure S13, a). The Bragg reflection at q≈15 nm^-1^ has index (100); in an ABO_3_ perovskite structure it is produced by A and B atoms (oxygen contribution is extinct). For the superstructure with Y atom occupying one corner in a 2x2x2 supercell, the reflex can be expected at q value of ½ of the (100) reflex. Normally, in the absence of superstructure, this reflex is extinct due to destructive interference from the scattering by every second atom, where scattered x-rays come in anti-phase. However, when the superstructure is present, the peak should appear due to the difference in the Sn and Y scattering factors (scattering contrast). Y and Sn have rather low contrast, both in neutron (Y: 7.75, Sn: 6.225) and conventional x-ray scattering (Y: 38.98, Sn: 49.99). Additionally, Y presumably has only a small stoichiometric fraction of 12.5%, so at normal scattering the peak is very weak (see for example Figure 1, b in the main text). The anomalous x-ray diffraction allows to enhance the contrast between the specific elements. Figure S13, b shows the anomalous correction f'(E) to the atomic scattering factors of yttrium and tin. At the energies close to the Y x-ray absorption edge at ~17000 eV the elastic scattering factor has a sharp drop, thus increasing the contrast between Y and Sn.


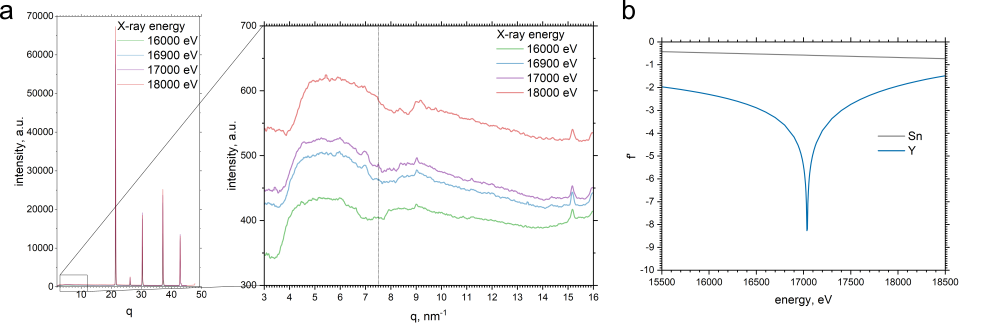


Figure S13 (a) Anomalous XRD, measured at various incident x-ray energies. (b) Anomalous correction to the atomic form-factor of Y and Sn atoms.

In the diffraction patterns at q=7.5 nm^-1^ the appearance of the peak is visible at the energies of incident photons of 16900 and 17000 eV, with the highest apparent intensity at 17000 eV, where the scattering factor of Y is the lowest and the contrast is the highest. While the intensity of this superstructure signature is relatively low in comparison to the principal peaks, it makes the calculations and the observed phase separation in a sample with net 10% Y content plausible for the presence of a superstructure in the Y-doped barium stannate.

**Proton conductivity in non-doped BaSnO_3_.**

We are confident that the ion transport quantified by impedance spectroscopy originates from protons and deuterons, respectively, because the conductivity follows the isotope effect [88]. This is because we measured the impedance with protonated and with deuterated samples under the same humidity concentration, and the conductivities yield a ratio of σH/σD ≈ 1.6. The relaxation times (time constants) differ only marginally, which is a manifestation that the isotope change affects predominantly the mass of the charge carrier and virtually not the vibration properties of the host lattice (compare Samgin, [89]).

**Table S3**: Conductivities and relaxation times of BaSnO_3_ based on proton conduction and deuteron conduction.

| Water vapor/gas | Conductivity σ [µS] | Relaxation time τ [s] |
| --- | --- | --- |
| H_2_O | 17.12 | 2.33 · 10^-11^ ± 0.02 · 10^-11^ |
| D_2_O | 10.71 | 2.37 · 10^-11^ ± 0.03 · 10^-11^ |

For comparison, Li and Nino [90] found for BaSnO_3_ a ratio of 2.6, and Nowick and Vaysley [91] compiled a list of proton conductors with perovskite structure where the ratio ranged between 1.5 and 4.0.

**Estimation of proton jump distance**

To estimate the change of O-O distance due to the thermal vibrations, we did the following:

Let us consider the proton jump between oxygen atoms along the edge of the oxygen octahedron. "Static" O-O distance *l* is:

$$l\left( O-O \right)=\frac{\sqrt{2}a}{2}$$

where a is the lattice parameter.

Since all phonon modes coexist simultaneously and contribute to the displacement of oxygen, we will not consider a specific phonon mode but averaged thermal displacement of oxygen atoms. According to the DFT modelling and Rietveld refinement of our experimental neutron diffraction data, the matrix element of the thermal displacement tensor U_11_ is significantly (~2-3 times) larger than U_33_. Let us therefore consider exclusively displacement perpendicular to Me-O-Me line along X axis in Figure S5. The root mean square displacement is a square root of a thermal displacement parameter, presented in Table S1; in the harmonic approximation the amplitude is:

$$\sqrt{2}*r.m.s. displacement= \sqrt{2}\sqrt{U_{11}}$$

From the geometry considerations, the change of the distance between two oxygen atoms due to thermal vibrations is:

$$\frac{2*\left\{ amplitude of displacement \right\}}{\sqrt{2}}=2\sqrt{U_{11}}$$

Calculated thermal displacement parameters fit the experimental ones, obtained from Rietveld refinement well and diverge by approx. 20% (at 200K: DFT calculated: BaSnO_3_: U_11_=0.0059 U_33_=0.0024; BaSn_7/8_Y_1/8_O_3_: U_11_=0.0070 U33=0.0027; for experimental values see Table S1); therefore, to estimate the value at typical operating temperature of 350 °C or ≈600 K, we will take values obtained from DFT calculations: 0.015 for BaSnO_3_ and 0.024 for BaSn_7/8_Y_1/8_O_3_.

Results are present in Table S4:

**Table S4**: Estimation of the change of O-O distance:

|  | Static O-O distance, Å | Amplitude of displacement at 600 K, Å | Min. O-O distance at 600 K, Å |
| --- | --- | --- | --- |
| BaSnO_3_ | 2.90858 | 0.17147 | 2.666089 |
| BaSn_7/8_Y_1/8_O_3_ | 2.93119 | 0.22527 | 2.612617 |
| difference | 0.022613 | 0.0538 | -0.05347 |

Considering that the O-H bond length is approximately 1 Å, the jump distance is ~0.9 Å, in "static" configuration, and is shorter by ~25% (0.25 Å) due to the thermal vibrations; in total, enhanced thermal vibrations of yttrium-doped sample make the jump distance ~10% shorter than in the undoped sample.

[1] A. Royant, K. Edman, T. Ursby, E. Pebay-Peyroula, E. M. Landau, R. Neutze, Helix deformation is coupled to vectorial proton transport in the photocycle of bacteriorhodopsin, *Nature* **2000**, *406* (6796), 645, <https://doi.org/10.1038/35020599>.

[2] P. Lipari, Proton and neutrino extragalactic astronomy, *Physical Review D* **2008**, *78* (8), <https://doi.org/10.1103/PhysRevD.78.083011>.

[3] A. Braun, *Electrochemical Energy Systems - Foundations, Energy Storage and Conversion*, Walter de Gruyter GmbH, Boston, Berlin **2019**.

[4] a) T. Norby, in *Fuel Cells and Hydrogen Energy*, **2009**, Ch. Chapter 11; b) K. D. Kreuer, in (Ed.: T. Ishihara), Springer, **2009**.

[5] K. D. Kreuer, Aspects of the formation and mobility of protonic charge carriers and the stability of perovskite-type oxides, *Solid State Ionics* **1999**, *125* (1-4), 285, <https://doi.org/10.1016/s0167-2738(99)00188-5>.

[6] Q. Chen, F. El Gabaly, F. A. Akgul, Z. Liu, B. S. Mun, S. Yamaguchi, A. Braun, Observation of Oxygen Vacancy Filling under Water Vapor in Ceramic Proton Conductors in Situ with Ambient Pressure XPS, *Chemistry of Materials* **2013**, *25* (23), 4690, <https://doi.org/10.1021/cm401977p>.

[7] a) T. S. Bjørheim, A. Løken, R. Haugsrud, On the relationship between chemical expansion and hydration thermodynamics of proton conducting perovskites, *Journal of Materials Chemistry A* **2016**, *4* (16), 5917, <https://doi.org/10.1039/c5ta10090a>; b) K. S. Knight, Structural phase transitions, oxygen vacancy ordering and protonation in doped BaCeO3: results from time-of-flight neutron powder diffraction investigations, *Solid State Ionics* **2001**, *145* (1-4), 275, <https://doi.org/10.1016/s0167-2738(01)00952-3>.

[8] D. Marx, Proton transfer 200 years after von Grotthuss: insights from ab initio simulations, *Chemphyschem* **2006**, *7* (9), 1848, <https://doi.org/10.1002/cphc.200600128>.

[9] W. Münch, Proton diffusion in perovskites: comparison between BaCeO3, BaZrO3, SrTiO3, and CaTiO3 using quantum molecular dynamics, *Solid State Ionics* **2000**, *136-137* (1-2), 183, <https://doi.org/10.1016/s0167-2738(00)00304-0>.

[10] a) T. Norby, M. Wideroe, R. Glockner, Y. Larring, Hydrogen in oxides, *Dalton Trans* **2004**, (19), 3012, <https://doi.org/10.1039/b403011g>; b) K. D. Kreuer, Proton conductivity: Materials and applications, *Chemistry of Materials* **1996**, *8* (3), 610, <https://doi.org/DOI> 10.1021/cm950192a.

[11] R. Hempelmann, *Quasielastic Neutron Scattering and Solid State Diffusion*, Oxford Science Publications, **2001**.

[12] a) C. Karmonik, R. Hempelmann, T. Matzke, T. Springer, Proton Diffusion in Strontium Cerate Ceramics Studied by Quasi-Elastic Neutron-Scattering and Impedance Spectroscopy, *Z Naturforsch A* **1995**, *50* (6), 539; b) R. Hempelmann, C. Karmonik, T. Matzke, M. Cappadonia, U. Stimming, T. Springer, M. A. Adams, Quasi-Elastic Neutron-Scattering Study of Proton Diffusion in SrCe0.95Yb0.05H0.02O2.985, *Solid State Ionics* **1995**, *77*, 152, <https://doi.org/Doi> 10.1016/0167-2738(94)00264-S; c) T. Matzke, U. Stimming, C. Karmonik, M. Soetratmo, R. Hempelmann, F. Guthoff, Quasielastic thermal neutron scattering experiment on the proton conductor SrCe0.95Yb0.05H0.02O2.985, *Solid State Ionics* **1996**, *86-8*, 621, <https://doi.org/10.1016/0167-2738(96)00223-8>.

[13] H. Niu, Y. Jing, Y. Sun, L. Guo, N. R. Aluru, W. Li, J. Yang, X. Li, On the anomalous diffusion of proton in Y-doped BaZrO3 perovskite oxide, *Solid State Ionics* **2022**, *376*, <https://doi.org/10.1016/j.ssi.2022.115859>.

[14] M. Karlsson, A. Matic, S. F. Parker, I. Ahmed, L. Börjesson, S. Eriksson, O−Hwag vibrations in hydratedBaInxZr1−xO3−x∕2investigated with inelastic neutron scattering, *Physical Review B* **2008**, *77* (10), <https://doi.org/10.1103/PhysRevB.77.104302>.

[15] A. Slodczyk, P. Colomban, S. Willemin, O. Lacroix, B. Sala, Indirect Raman identification of the proton insertion in the high-temperature [Ba/Sr][Zr/Ti]O3-modified perovskite protonic conductors, *Journal of Raman Spectroscopy* **2009**, *40* (5), 513, <https://doi.org/10.1002/jrs.2157>.

[16] a) Q. Chen, T.-W. Huang, M. Baldini, A. Hushur, V. Pomjakushin, S. Clark, W. L. Mao, M. H. Manghnani, A. Braun, T. Graule, Effect of Compressive Strain on the Raman Modes of the Dry and Hydrated BaCe0.8Y0.2O3Proton Conductor, *The Journal of Physical Chemistry C* **2011**, *115* (48), 24021, <https://doi.org/10.1021/jp208525j>; b) Q. Chen, A. Braun, A. Ovalle, C.-D. Savaniu, T. Graule, N. Bagdassarov, Hydrostatic pressure decreases the proton mobility in the hydrated BaZr0.9Y0.1O3 proton conductor, *Applied Physics Letters* **2010**, *97* (4), <https://doi.org/10.1063/1.3464162>; c) Q. Chen, S. Holdsworth, J. Embs, V. Pomjakushin, B. Frick, A. Braun, High-temperature high pressure cell for neutron-scattering studies, *High Pressure Research* **2012**, *32* (4), 471, <https://doi.org/10.1080/08957959.2012.725729>.

[17] A. Braun, Q. Chen, Experimental neutron scattering evidence for proton polaron in hydrated metal oxide proton conductors, *Nat Commun* **2017**, *8*, 15830, <https://doi.org/10.1038/ncomms15830>.

[18] A. L. Samgin, Lattice-assisted proton motion in perovskite oxides, *Solid State Ionics* **2000**, *136* (1-2), 291, <https://doi.org/Doi> 10.1016/S0167-2738(00)00406-9.

[19] S. B. C. Duval, *PhD Thesis*, Technische Universität München **2008**.

[20] Q. Chen, A. Braun, A. Ovalle, C.-D. Savaniu, T. Graule, N. Bagdassarov, Protons in lattice confinement: Static pressure on the Y-substituted, hydrated BaZrO3 ceramic proton conductor decreases proton mobility, *arXiv:1106.1091 [cond-mat.str-el]* **2011**, <https://doi.org/10.1063/1.3464162>.

[21] a) Q. Chen, J. Banyte, A. Braun, Influence of pellet pressure on structure and electrical conductivity on the BaCe0.9Y0.1O3-δ proton conductor, *Solid State Ionics* **2017**; b) Q. Chen, A. Braun, S. Yoon, N. Bagdassarov, T. Graule, Effect of lattice volume and compressive strain on the conductivity of BaCeY-oxide ceramic proton conductors, *Journal of the European Ceramic Society* **2011**, *31* (14), 2657, <https://doi.org/10.1016/j.jeurceramsoc.2011.02.014>.

[22] Z. Fan, N. Li, P. Du, W. Yang, Q. Chen, Influence of Lattice Dynamics on the Proton Transport in BaZrY-Oxide Perovskites under High Pressure, *The Journal of Physical Chemistry C* **2020**, *124* (41), 22376, <https://doi.org/10.1021/acs.jpcc.0c06463>.

[23] H. Wang, E. Alp, Y. Yoda, S. P. Cramer, in (Ed.: J. C. Fontecilla-Camps), Clifton, N.J., **2014**.

[24] a) S. P. Cramer, Y. Xiao, H. Wang, Y. Guo, M. C. Smith, Nuclear Resonance Vibrational Spectroscopy (NRVS) of Fe–S model compounds, Fe–S proteins, and nitrogenase, *Hyperfine Interactions* **2006**, *170* (1-3), 47, <https://doi.org/10.1007/s10751-006-9473-4>; b) Y. M. Xiao, K. Fisher, M. C. Smith, W. E. Newton, D. A. Case, S. J. George, H. X. Wang, W. Sturhahn, E. E. Alp, J. Y. Zhao, Y. Yoda, S. P. Cramer, How nitrogenase shakes - Initial information about P-cluster and FeMo-cofactor normal modes from nuclear resonance vibrational Spectroscopy (NRVS), *Journal of the American Chemical Society* **2006**, *128* (23), 7608, <https://doi.org/10.1021/ja0603655>.

[25] J. Rehman, M. B. Hanif, M. Z. Khan, M. Ullah, I. A. Starostina, M. T. Muhammad, Z. Li, A Review of Proton-Conducting Electrolytes for Efficient Low-Temperature Solid Oxide Fuel Cells: Progress, Challenges, and Perspectives, *Energy & Fuels* **2024**, *38* (23), 22637, <https://doi.org/10.1021/acs.energyfuels.4c03683>.

[26] C. Malica, A. Dal Corso, Temperature-dependent atomic B factor: an ab initio calculation, *Acta Crystallogr A Found Adv* **2019**, *75* (Pt 4), 624, <https://doi.org/10.1107/S205327331900514X>.

[27] D. Phelan, F. Han, A. Lopez-Bezanilla, M. J. Krogstad, Y. Gim, Y. Rong, J. Zhang, D. Parshall, H. Zheng, S. L. Cooper, M. Feygenson, W. Yang, Y.-S. Chen, Structural properties of barium stannate, *Journal of Solid State Chemistry* **2018**, *262*, 142, <https://doi.org/10.1016/j.jssc.2018.01.019>.

[28] a) I. A. Alagdal, A. R. West, Oxygen stoichiometry, conductivity and gas sensing properties of BaSnO3, *J Mater Chem C* **2016**, *4* (21), 4770, <https://doi.org/10.1039/c6tc01007e>; b) J. Cui, Y. Zhang, J. Wang, Z. Zhao, H. Huang, W. Zou, M. Yang, R. Peng, W. Yan, Q. Huang, Z. Fu, Y. Lu, Oxygen deficiency induced strong electron localization in lanthanum doped transparent perovskite oxide BaSnO3, *Physical Review B* **2019**, *100* (16), <https://doi.org/10.1103/PhysRevB.100.165312>; c) H. M. I. Jaim, S. Lee, X. Zhang, I. Takeuchi, Stability of the oxygen vacancy induced conductivity in BaSnO3 thin films on SrTiO3, *Applied Physics Letters* **2017**, *111* (17), <https://doi.org/10.1063/1.4996548>.

[29] E. Moreira, J. M. Henriques, D. L. Azevedo, E. W. S. Caetano, V. N. Freire, E. L. Albuquerque, Structural and electronic properties of SrxBa1−xSnO3 from first principles calculations, *Journal of Solid State Chemistry* **2012**, *187*, 186, <https://doi.org/10.1016/j.jssc.2011.12.027>.

[30] A. Braun, A. Ovalle, V. Pomjakushin, A. Cervellino, S. Erat, W. C. Stolte, T. Graule, Yttrium and hydrogen superstructure and correlation of lattice expansion and proton conductivity in the BaZr0.9Y0.1O2.95 proton conductor, *Applied Physics Letters* **2009**, *95* (22), <https://doi.org/10.1063/1.3268454>.

[31] F. M. Draber, C. Ader, J. P. Arnold, S. Eisele, S. Grieshammer, S. Yamaguchi, M. Martin, Nanoscale percolation in doped BaZrO(3) for high proton mobility, *Nat Mater* **2020**, *19* (3), 338, <https://doi.org/10.1038/s41563-019-0561-7>.

[32] E. Fransson, P. Rosander, P. Erhart, G. Wahnström, Understanding Correlations in BaZrO3: Structure and Dynamics on the Nanoscale, *Chemistry of Materials* **2023**, *36* (1), 514, <https://doi.org/10.1021/acs.chemmater.3c02548>.

[33] I. A. Zvonareva, G. N. Starostin, M. T. Akopian, G. K. Vdovin, X.-Z. Fu, D. A. Medvedev, Ionic and electronic transport of dense Y-doped barium stannate ceramics for high-temperature applications, *Journal of Power Sources* **2023**, *565*, <https://doi.org/10.1016/j.jpowsour.2023.232883>.

[34] H. Wang, A. Braun, S. P. Cramer, L. B. Gee, Y. Yoda, Nuclear Resonance Vibrational Spectroscopy: A Modern Tool to Pinpoint Site-Specific Cooperative Processes, *Catalysts* **2021**, *11* (8), <https://doi.org/10.3390/cryst11080909>.

[35] a) A. Rulev, H. Wang, S. Erat, M. Aycibin, D. Rentsch, V. Pomjakushin, S. P. Cramer, Q. Chen, N. Nagasawa, Y. Yoda, A. Braun, 119Sn Element-Specific Phonon Density of States of BaSnO3, *Crystals* **2025**, *15* (5), <https://doi.org/10.3390/cryst15050440>; b) A. Rulev, N. Nagasawa, H. Li, H. Wang, S. P. Cramer, Q. Chen, Y. Yoda, A. Braun, Operando NRVS on LiFePO4 Battery with 57Fe Phonon DOS, *Crystals* **2025**, *15* (10), <https://doi.org/10.3390/cryst15100841>; c) A. Rulev, N. Nagasawa, H. Li, Q. Chen, H. Wang, S. P. Cramer, Y. Yoda, A. Braun, The Fe projected phonon density of states in the LiFePO4 battery during operation: an electro-chemical operando nuclear resonant vibration spectroscopy study, *Crystals* **2025**, *in preparation*.

[36] a) A. Braun, *X-ray Studies on Electrochemical Systems - Synchrotron Methods for Energy Materials*, Walter De Gruyter GmbH, Berlin/Boston **2017**; b) A. Braun, *X-Ray Studies on Electrochemical Systems - Synchrotron Methods for Energy Materials*, Walter De Gruyter GmbH, **2024**.

[37] C. Y. Regalado Vera, H. Ding, J. Urban-Klaehn, M. Li, Z. Zhao, F. Stewart, H. Tian, X. Liu, Y. Dong, J. Li, M. Zhou, H. Luo, D. Ding, Improving Proton Conductivity by Navigating Proton Trapping in High Scandium-Doped Barium Zirconate Electrolytes, *Chemistry of Materials* **2023**, *35* (14), 5341, <https://doi.org/10.1021/acs.chemmater.3c00531>.

[38] a) Y. Yamazaki, F. Blanc, Y. Okuyama, L. Buannic, J. C. Lucio-Vega, C. P. Grey, S. M. Haile, Proton trapping in yttrium-doped barium zirconate, *Nat Mater* **2013**, *12* (7), 647, <https://doi.org/10.1038/nmat3638>; b) R. Hempelmann, Quasielastic neutron scattering study of proton diffusion in SrCe0.95Yb0.05H0.02O2.985, *Solid State Ionics* **1995**, *77*, 152, <https://doi.org/10.1016/0167-2738(94)00264-s>.

[39] J. L. Bao, D. G. Truhlar, Variational transition state theory: theoretical framework and recent developments, *Chem Soc Rev* **2017**, *46* (24), 7548, <https://doi.org/10.1039/c7cs00602k>.

[40] Y. Wang, A. Chesnaud, E. Bevillon, G. Dezanneau, Properties of Y-doped BaSnO3 proton conductors, *Solid State Ionics* **2012**, *214*, 45, <https://doi.org/10.1016/j.ssi.2012.02.045>.

[41] a) P. G. Sundell, M. E. Björketun, G. Wahnström, Density-functional calculations of prefactors and activation energies for H diffusion inBaZrO3, *Physical Review B* **2007**, *76* (9), <https://doi.org/10.1103/PhysRevB.76.094301>; b) M. E. Björketun, P. G. Sundell, G. Wahnström, Effect of acceptor dopants on the proton mobility inBaZrO3: A density functional investigation, *Physical Review B* **2007**, *76* (5), <https://doi.org/10.1103/PhysRevB.76.054307>; c) M. Bjorketun, P. Sundell, G. Wahnstrom, D. Engberg, A kinetic Monte Carlo study of proton diffusion in disordered perovskite structured lattices based on first-principles calculations, *Solid State Ionics* **2005**, *176* (39-40), 3035, <https://doi.org/10.1016/j.ssi.2005.09.044>; d) M. K. Hossain, M. C. Biswas, R. K. Chanda, M. H. K. Rubel, M. I. Khan, K. Hashizume, **2021**, <https://doi.org/10.21203/rs.3.rs-397968/v1>.

[42] E. Libowitzky, in (Eds.: P. Schuster, W. Mikenda), Springer, Vienna **1999**.

[43] G. H. Vineyard, Frequency factors and isotope effects in solid state rate processes, *Journal of Physics and Chemistry of Solids* **1957**, *3* (1-2), 121, <https://doi.org/10.1016/0022-3697(57)90059-8>.

[44] M. T. Dove, *Introduction to Lattice Dynamics*, **2010**.

[45] V. M. Goldschmidt, Die Gesetze der Krystallochemie, *Die Naturwissenschaften* **1926**, *14* (21), 477, <https://doi.org/10.1007/BF01507527>.

[46] C. J. Bartel, C. Sutton, B. R. Goldsmith, R. Ouyang, C. B. Musgrave, L. M. Ghiringhelli, M. Scheffler, New tolerance factor to predict the stability of perovskite oxides and halides, *Sci Adv* **2019**, *5* (2), eaav0693, <https://doi.org/10.1126/sciadv.aav0693>.

[47] L. Malavasi, C. Ritter, G. Chiodelli, Correlation between Thermal Properties, Electrical Conductivity, and Crystal Structure in the BaCe0.80Y0.20O2.9 Proton Conductor, *Chemistry of Materials* **2008**, *20* (6), 2343, <https://doi.org/10.1021/cm7033917>.

[48] C. H. Schwalbe, Lars Vegard: key communicator and pioneer crystallographer, *Crystallography Reviews* **2014**, *20* (1), 9, <https://doi.org/10.1080/0889311x.2013.838674>.

[49] a) K. Kamata, T. Nakamura, T. Sata, On the State of d-electrons in perovskite-type compounds ABO3, *Bulletin of Tokyo Institute of Technology* **1974**, *120*, 73; b) K. Kamata, T. Nakamura, Graphical Discrimination of Localized or Itinerant Electrons in Perovskite Compounds ABO3, *Journal of the Physical Society of Japan* **1973**, *35* (5), 1558, <https://doi.org/10.1143/jpsj.35.1558>.

[50] R. D. Shannon, Revised effective ionic radii and systematic studies of interatomic distances in halides and chalcogenides, *Acta Crystallographica Section A* **1976**, *32* (5), 751, <https://doi.org/10.1107/s0567739476001551>.

[51] A. M. Glazer, The classification of tilted octahedra in perovskites, *Acta Crystallographica Section B Structural Crystallography and Crystal Chemistry* **1972**, *28* (11), 3384, <https://doi.org/10.1107/s0567740872007976>.

[52] J.-W. Jhuang, K.-R. Lee, S.-W. Lee, J.-K. Chang, S.-C. Jang, C.-J. Tseng, Microstructures and electrical properties of zirconium doped barium cerate perovskite proton conductors, *International Journal of Hydrogen Energy* **2019**, *44* (38), 21174, <https://doi.org/10.1016/j.ijhydene.2019.04.185>.

[53] A. Togo, I. Tanaka, First principles phonon calculations in materials science, *Scripta Materialia* **2015**, *108*, 1, <https://doi.org/10.1016/j.scriptamat.2015.07.021>.

[54] I. Pallikara, P. Kayastha, J. M. Skelton, L. D. Whalley, The physical significance of imaginary phonon modes in crystals, *Electronic Structure* **2022**, *4* (3), <https://doi.org/10.1088/2516-1075/ac78b3>.

[55] J. Richter, P. Holtappels, T. Graule, T. Nakamura, L. J. Gauckler, Materials design for perovskite SOFC cathodes, *Monatsh. Chem.* **2009**, *140* (9), 985, <https://doi.org/10.1007/s00706-009-0153-3>.

[56] N. K. Nepal, P. C. Canfield, L.-L. Wang, Imaginary phonon modes and phonon-mediated superconductivity in Y2C3, *Physical Review B* **2024**, *109* (5), <https://doi.org/10.1103/PhysRevB.109.054518>.

[57] G. Krenzer, C.-E. Kim, K. Tolborg, B. J. Morgan, A. Walsh, Anharmonic lattice dynamics of superionic lithium nitride, *Journal of Materials Chemistry A* **2022**, *10* (5), 2295, <https://doi.org/10.1039/d1ta07631k>.

[58] M. K. Gupta, J. Ding, N. C. Osti, D. L. Abernathy, W. Arnold, H. Wang, Z. Hood, O. Delaire, Fast Na diffusion and anharmonic phonon dynamics in superionic Na3PS4, *Energy & Environmental Science* **2021**, *14* (12), 6554, <https://doi.org/10.1039/d1ee01509e>.

[59] M. K. Gupta, S. Kumar, R. Mittal, S. L. Chaplot, Soft-phonon anharmonicity, floppy modes, and Na diffusion in Na3FY (Y=S,Se,Te): Ab initio and machine-learned molecular dynamics simulations, *Physical Review B* **2022**, *106* (1), <https://doi.org/10.1103/PhysRevB.106.014311>.

[60] M. K. Gupta, J. Ding, D. Bansal, D. L. Abernathy, G. Ehlers, N. C. Osti, W. G. Zeier, O. Delaire, Strongly Anharmonic Phonons and Their Role in Superionic Diffusion and Ultralow Thermal Conductivity of Cu7PSe6, *Advanced Energy Materials* **2022**, *12* (23), <https://doi.org/10.1002/aenm.202200596>.

[61] A. I. Lebedev, I. A. Sluchinskaya, Structural instability in BaZrO3 crystals: Calculations and experiment, *Physics of the Solid State* **2013**, *55* (9), 1941, <https://doi.org/10.1134/s1063783413090229>.

[62] S. Imashuku, T. Uda, Y. Nose, Y. Awakura, Effect of isovalent cation substitution on conductivity and microstructure of sintered yttrium-doped barium zirconate, *Journal of Alloys and Compounds* **2010**, *490* (1-2), 672, <https://doi.org/10.1016/j.jallcom.2009.10.135>.

[63] Q. L. Chen, A. Braun, S. Yoon, N. Bagdassarov, T. Graule, Effect of lattice volume and compressive strain on the conductivity of BaCeY-oxide ceramic proton conductors, *Journal of the European Ceramic Society* **2011**, *31* (14), 2657, <https://doi.org/10.1016/j.jeurceramsoc.2011.02.014>.

[64] W. Münch, G. Seifert, K. D. Kreuer, J. Maier, A quantum molecular dynamics study of proton conduction phenomena in BaCeO3, *Solid State Ionics* **1996**, *86-88*, 647, <https://doi.org/10.1016/0167-2738(96)00229-9>.

[65] K. Kreuer, H/D isotope effect of proton conductivity and proton conduction mechanism in oxides, *Solid State Ionics* **1995**, *77*, 157, <https://doi.org/10.1016/0167-2738(94)00265-t>.

[66] H. Zhou, Y. Ji, Y. Wang, K. Feng, B. Luan, X. Zhang, L.-Q. Chen, First-principles lattice dynamics and thermodynamic properties of α-, θ-, κ- and γ-Al2O3 and solid state temperature-pressure phase diagram, *Acta Materialia* **2024**, *263*, <https://doi.org/10.1016/j.actamat.2023.119513>.

[67] a) K. Wakamura, Empirical relationships for ion conduction based on vibration amplitude in perovskite-type proton and superionic conductors, *Journal of Physics and Chemistry of Solids* **2005**, *66* (1), 133, <https://doi.org/10.1016/j.jpcs.2004.08.044>; b) K. Wakamura, Ion conduction in proton- and related defect (super) ionic conductors: Mechanical, electronic and structure parameters, *Solid State Ionics* **2009**, *180* (26-27), 1343, <https://doi.org/10.1016/j.ssi.2009.08.009>.

[68] T. Song, Y. X. Lin, D. Wang, Q. L. Chen, C. Ling, S. Q. Shi, Renewing Fundamental Understanding of Ionic Transport in Inorganic Crystalline Solid-State Electrolytes from the Perspective of Lattice Dynamics, *Advanced Energy Materials* **2024**, <https://doi.org/10.1002/aenm.202302440>.

[69] S. Muy, R. Schlem, Y. Shao‐Horn, W. G. Zeier, Phonon–Ion Interactions: Designing Ion Mobility Based on Lattice Dynamics, *Advanced Energy Materials* **2020**, *11* (15), <https://doi.org/10.1002/aenm.202002787>.

[70] a) G. S. Bauer, S. O. C. European Nucl, SINQ as a versatile alternative neutron source, *Rrfm'99: 3rd International Topical Meeting on Research Reactor Fuel Management* **1999**, 1; b) P. Fischer, G. Frey, M. Koch, M. Könnecke, V. Pomjakushin, J. Schefer, R. Thut, N. Schlumpf, R. Bürge, U. Greuter, S. Bondt, E. Berruyer, High-resolution powder diffractometer HRPT for thermal neutrons at SINQ, *Physica B: Condensed Matter* **2000**, *276-278*, 146, <https://doi.org/10.1016/s0921-4526(99)01399-x>; c) W. E. Fischer, SINQ - The spallation neutron source, a new research facility at PSI, *Physica B* **1997**, *234*, 1202, <https://doi.org/10.1016/s0921-4526(97)00260-3>.

[71] A. Braun, S. Duval, P. Ried, J. Embs, F. Juranyi, T. Straessle, U. Stimming, R. Hempelmann, P. Holtappels, T. Graule, Proton diffusivity in the BaZr0.9Y0.1O3-delta proton conductor, *Journal of Applied Electrochemistry* **2009**, *39* (4), 471, <https://doi.org/10.1007/s10800-008-9667-3>.

[72] K. Kobayashi, Y. Sakka, T. S. Suzuki, Development of an electrochemical impedance analysis program based on the expanded measurement model, *Journal of the Ceramic Society of Japan* **2016**, *124* (9), 943, <https://doi.org/10.2109/jcersj2.16120>.

[73] N. S. Goroff, IGOR Pro, Version 3 (Mac) Wavemetrics, Inc.:  P.O. Box 2088, Lake Oswego, Oregon, 97035. Phone:  (503) 620-3001. Fax:  (503) 620-6754. E-mail:  sales@wavemetrics.com, <http://www.wavemetrics.com>. List Price $495.00; students and faculty $346.50; additional quantity, multi-user, and coursework discounts available, *Journal of the American Chemical Society* **1997**, *119* (43), 10567, <https://doi.org/10.1021/ja965932a>.

[74] a) F. J. A. Loureiro, Z. Shakel, V. C. D. Graça, L. I. V. Holz, D. P. Fagg, Benchmarking the yttrium content in the low temperature/low humidity electrical properties of yttrium-doped barium cerate, *Ceramics International* **2023**, *49* (21), 34303, <https://doi.org/10.1016/j.ceramint.2023.08.148>; b) I. A. Starostina, G. N. Starostin, M. T. Akopian, G. K. Vdovin, D. A. Osinkin, B. Py, A. Maradesa, F. Ciucci, D. A. Medvedev, Insight into Grain and Grain‐Boundary Transport of Proton‐Conducting Ceramics: A Case Report of BaSn0.8Y0.2O3−δ, *Advanced Functional Materials* **2023**, *34* (6), <https://doi.org/10.1002/adfm.202307316>.

[75] a) A. Q. R. Baron, Y. Tanaka, D. Miwa, D. Ishikawa, T. Mochizuki, K. Takeshita, S. Goto, T. Matsushita, H. Kimura, F. Yamamoto, T. Ishikawa, Early commissioning of the SPring-8 beamline for high resolution inelastic X-ray scattering, *Nuclear Instruments and Methods in Physics Research Section A: Accelerators, Spectrometers, Detectors and Associated Equipment* **2001**, *467-468*, 627, <https://doi.org/10.1016/s0168-9002(01)00431-4>; b) Y. Yoda, X-ray beam properties available at the nuclear resonant scattering beamline at SPring-8, *Hyperfine Interactions* **2019**, *240* (1), <https://doi.org/10.1007/s10751-019-1598-3>.

[76] L. B. Gee, H. Wang, S. P. Cramer, NRVS for Fe in Biology: Experiment and Basic Interpretation, *Methods Enzymol.* **2018**, *599*, 409, <https://doi.org/10.1016/bs.mie.2017.11.002>.

[77] W. Sturhahn, CONUSS and PHOENIX: Evaluation of nuclear resonant scattering data, *Hyperfine Interactions* **2000**, *125* (1/4), 149, <https://doi.org/10.1023/a:1012681503686>.

[78] H. Yang, C. Hu, Y. Zhou, X. Liu, Y. Shi, J. Li, G. Li, Z. Chen, S. Chen, C. Zeni, M. Horton, R. Pinsler, A. Fowler, D. Zügner, T. Xie, J. Smith, L. Sun, Q. Wang, L. Kong, C. Liu, H. Hao, Z. Lu, MatterSim: A Deep Learning Atomistic Model Across Elements, Temperatures and Pressures. **2024**; p arXiv:2405.04967.

[79] a) P. Giannozzi, S. Baroni, N. Bonini, M. Calandra, R. Car, C. Cavazzoni, D. Ceresoli, G. L. Chiarotti, M. Cococcioni, I. Dabo, A. Dal Corso, S. de Gironcoli, S. Fabris, G. Fratesi, R. Gebauer, U. Gerstmann, C. Gougoussis, A. Kokalj, M. Lazzeri, L. Martin-Samos, N. Marzari, F. Mauri, R. Mazzarello, S. Paolini, A. Pasquarello, L. Paulatto, C. Sbraccia, S. Scandolo, G. Sclauzero, A. P. Seitsonen, A. Smogunov, P. Umari, R. M. Wentzcovitch, QUANTUM ESPRESSO: a modular and open-source software project for quantum simulations of materials, *J Phys Condens Matter* **2009**, *21* (39), 395502, <https://doi.org/10.1088/0953-8984/21/39/395502>; b) P. Giannozzi, O. Andreussi, T. Brumme, O. Bunau, M. Buongiorno Nardelli, M. Calandra, R. Car, C. Cavazzoni, D. Ceresoli, M. Cococcioni, N. Colonna, I. Carnimeo, A. Dal Corso, S. de Gironcoli, P. Delugas, R. A. DiStasio, Jr., A. Ferretti, A. Floris, G. Fratesi, G. Fugallo, R. Gebauer, U. Gerstmann, F. Giustino, T. Gorni, J. Jia, M. Kawamura, H. Y. Ko, A. Kokalj, E. Kucukbenli, M. Lazzeri, M. Marsili, N. Marzari, F. Mauri, N. L. Nguyen, H. V. Nguyen, A. Otero-de-la-Roza, L. Paulatto, S. Ponce, D. Rocca, R. Sabatini, B. Santra, M. Schlipf, A. P. Seitsonen, A. Smogunov, I. Timrov, T. Thonhauser, P. Umari, N. Vast, X. Wu, S. Baroni, Advanced capabilities for materials modelling with Quantum ESPRESSO, *J Phys Condens Matter* **2017**, *29* (46), 465901, <https://doi.org/10.1088/1361-648X/aa8f79>; c) P. Giannozzi, O. Baseggio, P. Bonfa, D. Brunato, R. Car, I. Carnimeo, C. Cavazzoni, S. de Gironcoli, P. Delugas, F. Ferrari Ruffino, A. Ferretti, N. Marzari, I. Timrov, A. Urru, S. Baroni, Quantum ESPRESSO toward the exascale, *J Chem Phys* **2020**, *152* (15), 154105, <https://doi.org/10.1063/5.0005082>.

[80] J. P. Perdew, K. Burke, M. Ernzerhof, Generalized Gradient Approximation Made Simple, *Phys Rev Lett* **1996**, *77* (18), 3865, <https://doi.org/10.1103/PhysRevLett.77.3865>.

[81] G. Prandini, A. Marrazzo, I. E. Castelli, N. Mounet, N. Marzari, Precision and efficiency in solid-state pseudopotential calculations, *npj Computational Materials* **2018**, *4* (1), <https://doi.org/10.1038/s41524-018-0127-2>.

[82] N. E. Kirchner-Hall, W. Zhao, Y. Xiong, I. Timrov, I. Dabo, Extensive Benchmarking of DFT+U Calculations for Predicting Band Gaps, *Applied Sciences* **2021**, *11* (5), <https://doi.org/10.3390/app11052395>.

[83] a) A. Togo, L. Chaput, T. Tadano, I. Tanaka, Implementation strategies in phonopy and phono3py, *J Phys Condens Matter* **2023**, *35* (35), <https://doi.org/10.1088/1361-648X/acd831>; b) A. Togo, First-principles Phonon Calculations with Phonopy and Phono3py, *Journal of the Physical Society of Japan* **2023**, *92* (1), <https://doi.org/10.7566/jpsj.92.012001>.

[84] W. Aggoune, A. Eljarrat, D. Nabok, K. Irmscher, M. Zupancic, Z. Galazka, M. Albrecht, C. Koch, C. Draxl, A consistent picture of excitations in cubic BaSnO3 revealed by combining theory and experiment, *Communications Materials* **2022**, *3* (1), <https://doi.org/10.1038/s43246-022-00234-6>.

[85] Y. Ikeda, A. Carreras, A. Seko, A. Togo, I. Tanaka, Mode decomposition based on crystallographic symmetry in the band-unfolding method, *Physical Review B* **2017**, *95* (2), <https://doi.org/10.1103/PhysRevB.95.024305>.

[86] A. Rulev, MatterSim fine-tuned MLIP for BaSnO₃ and Y-doped BaSnO₃ perovskites with oxygen vacancies (10.5281/zenodo.17407327). A. Rulev, Ed.: Zenodo: Dübendorf, **2025**.

[87] Y. Waseda, *Anomalous X-Ray Scattering for Material Characterization - Atomic-Scale Structure Determination*, Springer, Berlin, Heidelberg **2002**.

[88] G. Jancsó, in (Eds.: A. Vértes, S. Nagy, Z. Klencsár, R. G. Lovas, F. Rösch), Springer, Boston MA **2011**, Ch. Chapter 15.

[89] A. Samgin, A statistical theory of the isotope effect in proton conducting oxides, *Solid State Ionics* **2005**, *176* (23-24), 1837, <https://doi.org/10.1016/j.ssi.2005.05.007>.

[90] L. Li, J. C. Nino, Proton-conducting barium stannates: Doping strategies and transport properties, *International Journal of Hydrogen Energy* **2013**, *38* (3), 1598, <https://doi.org/10.1016/j.ijhydene.2012.11.065>.

[91] A. S. Nowick, A. V. Vaysleyb, Isotope effect and proton hopping in high-temperature protonic conductors, *Solid State Ionics* **1997**, *97* (1-4), 17, <https://doi.org/10.1016/s0167-2738(97)00081-7>.
